# Oxidation Camouflages Terrestrial Organic Matter to Appear Marine-like

**DOI:** 10.1021/acs.est.4c12913

**Published:** 2025-03-14

**Authors:** Aleksandar I. Goranov, Susan J. Carter, Ann Pearson, Patrick G. Hatcher

**Affiliations:** a Department of Chemistry and Biochemistry, Old Dominion University, Norfolk, Virginia 23529, United States; b Department of Earth and Planetary Sciences, Harvard University, Cambridge, Massachusetts 02138, United States

**Keywords:** oxidation, terrestrial carbon, global carbon cycle, isotopic fractionation

## Abstract

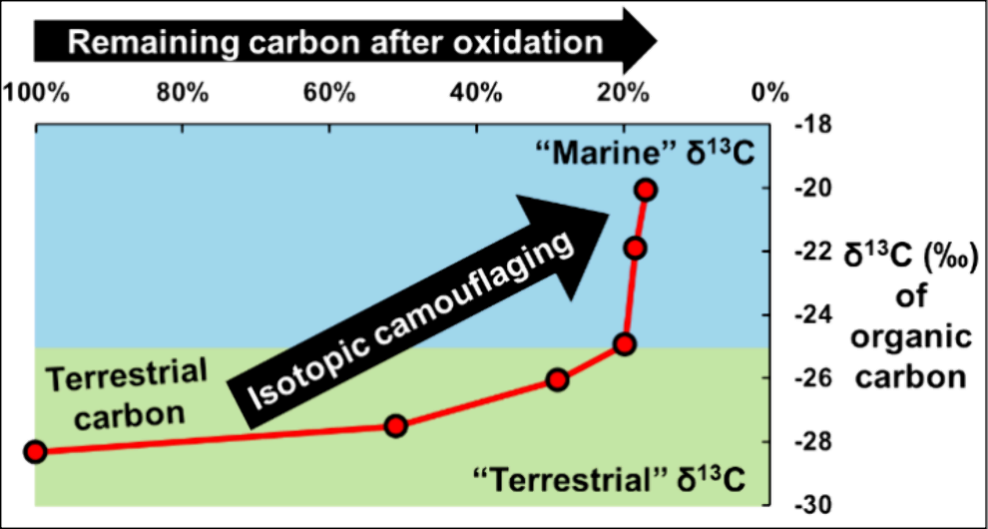

The export of terrestrial organic matter (TOM) to the ocean has been traditionally viewed to be minimal or only of significance in near-coastal continental margins. The broadly accepted explanation for the widespread loss of terrestrial lignin biomarkers and apparent disappearance of the <−25‰ stable carbon isotopic signature (δ^13^C) of TOM is that TOM is almost fully degraded before reaching the open ocean. Oceanic carbon (δ^13^C value ≥ −22‰) is therefore thought to be derived primarily from algae. However, an alternative explanation for the different molecular and δ^13^C signatures in terrestrial and marine environments may be that oxidative processing transforms TOM to appear marine-like. To test this hypothesis, we subjected eight TOM samples to a strong oxidation gradient. At carbon losses typical of TOM degradation during transport and sedimentation (above 60%), the differentiators of provenance disappeared, leaving a residue that was marine-like both chemically (aliphatic- and nitrogen-rich) and isotopically (δ^13^C enrichment of 4 to 9‰). This challenges the validity of conventional two-endmember mixing models, suggesting that a much larger fraction than previously estimated of the organic matter found in the ocean may originate from terrestrial sources, impacting global models of carbon cycling and sequestration.

## Abstract 2

Terrestrial aromatic-rich organic matter (δ^13^C < −25‰) is oxidized into marine-like aliphatic molecules (δ^13^C ≥ −22‰), implying that oceanic carbon likely has higher contributions from terrestrial sources than previously recognized.

## 1 Introduction

Terrestrial organic matter (TOM) plays a crucial role in the global carbon cycle. It is a complex mixture of fresh and humified biomacromolecules derived from vascular plants, soil organic matter, charcoalified materials, and microbial necromass. Each year, riverine and aeolian processes laterally export approximately 2.90 Pg of TOM-carbon (10^15^ g, Pg-C).^1^ During fluvial transport, TOM is biogeochemically processed, and 0.60 Pg-C are buried into freshwater sediments (flocculation/aggregation processes), while 2.12 Pg-C are outgassed into the atmosphere as CO_2_, CH_4_, or other volatile species.^1−3^ To the 2.90 Pg-C of the TOM flux, about 0.3 Pg-C are added from aquatic photosynthesis.^4^ Following fluvial processing by biotic and abiotic degradation processes (e.g., microbial respiration, enzymatic degradation, and abiotic photochemical and/or Fenton oxidation), about 0.48 Pg-C of TOM remain and are exported to oceanic reservoirs.^5^ This flux is more than 3 times larger than the annual oceanic burial of organic matter (0.15 Pg-C),^6^ and thus, it can supply all of the organic carbon buried in marine sediments while also sustaining the turnover of dissolved oceanic carbon.^5,7−11^ Despite this substantial flux, the elemental, structural, and carbon isotopic (δ^13^C) signatures of the TOM nearly vanish during the land–ocean transition. This has led to the prevailing idea that minimal TOM survives into marine systems;^8,11−13^ i.e., nearly all the residual 0.48 Pg-C^5^ TOM have been further quantitatively outgassed into the atmosphere during estuarine and surface ocean processing.

Oceanic organic matter (OOM) is therefore believed to originate primarily from *in situ* oceanic sources, mainly phytoplankton (i.e., algae), which fix atmospheric CO_2_ and sequester OOM through the biological pump. Quantitatively, TOM is viewed to contribute minimally to 700 Pg-C of dissolved OOM or 1750 Pg-C of buried sedimentary OOM.^14^ The annual sedimentary burial of 0.15 Pg-C^6^ is therefore believed to represent the degraded remains of marine microbes and their exudates (0.3% preservation of 50 Pg-C).^15^ This notion arises despite the high degradability of phytoplankton cells and their lack of recalcitrant biopolymers, such as lignin and cutin (although some microbes do contain algaenan or chitin). Previous estimates suggest that less than 1% of primary production escapes the degradation in the water column to settle in sediments,^16^ and most of the OOM surviving export is unrecognizable to conventional molecular characterization methods.^17^ Thus, dissolved, particulate, or sedimentary OOM pools are usually described as being comprised of highly degraded material of predominantly phytoplanktonic origin.^10^

Distinctions based on δ^13^C values, aliphatic versus aromatic character, lignin phenol content, and carbon-to-nitrogen (C/N) ratios collectively have established endmember criteria for estimating the contributions of TOM and OOM to oceanic systems.^11^ The aliphatic richness of OOM contrasts with the highly aromatic character of TOM, which mainly originates from plant-derived lignin.^18^ The aliphatic entities characteristic of OOM may, in contrast, come from a variety of sources. The melanoidin theory proposes that sugars and proteins, prevalent in marine algae, undergo polymerization in the water column and sediments to form recalcitrant, relatively non-aromatic macromolecules,^19^ perhaps originating from catalytic reactions on mineral surfaces that promote random intermolecular reactions.^20^ Selective preservation of biopolymeric components of primary production, such as algaenan, also may create highly branched and lipid-rich but relatively non-aromatic complex structures.^21−23^

Carbon stable isotope signatures (δ^13^C) and molar C/N ratios provide additional distinguishing criteria.^24−28^ The global average δ^13^C value of C_3_ plant biomass is −28.5‰.^29^ Even in riverine basins draining regions with significant C_4_ plant production, the total particulate organic matter in the river system often shows minimal quantitative contribution from the ^13^C-enriched C_4_ pathway (e.g., ≈−25‰ in the Mississippi River).^30,31^ In contrast, the δ^13^C values of fresh marine phytoplankton biomass range −20 to −22‰, closely resembling the values found in dissolved and particulate (i.e., suspended/sinking) marine OOM.^32^ Consequently, marine samples yielding δ^13^C values ≥ −22‰ are regarded as fully marine in origin. OOM samples also typically exhibit relatively high nitrogen content (i.e., C/N ratios ranging 6–8), similar to phytoplanktonic OOM, in contrast to the nitrogen-poor nature of fresh TOM (C/N ratios >10).^18,33^

These distinctive attributes have informed studies of both modern and ancient depositional systems. In regions like the Amazon River confluence with the South Atlantic Ocean,^34^ the discharge of the Yangtze River in China into the South China Sea,^35^ the outflow of the Congo River into modern^36,37^ and ancient^38^ lobes of the Congo submarine canyon, the Angola Basin,^39^ the Mississippi River delta,^40,41^ and the Washington Margin,^18^ sediments near river mouths are rich in compounds representing the TOM endmember and gradually transition towards the marine/algal characteristics of OOM offshore beyond the shelf break. This apparent mixing of TOM and OOM is supported by δ^13^C studies demonstrating a seaward progression from terrestrial-like isotopic compositions to more marine-like signatures.^11,18^ Thus, the changes observed in chemical properties (most notably, in bulk δ^13^C signatures) across land-to-ocean continua have been simply explained by pure mixing models, such as the one shown in eq 1 for bulk δ^13^C signatures (*f*_TOM_ and *f*_OOM_ denoting the fractions of TOM and OOM, respectively). This widely accepted two-endmember mixing hypothesis has been founded on the idea that the δ^13^C values of TOM (δ^13^C_TOM_ typically set ≈−29‰) and OOM (δ^13^C_OOM_ typically set ≈−22‰) are insignificantly changed during biogeochemical processing such as biotic (aerobic respiration, enzymatic oxidation) or abiotic oxidation (e.g., photochemical/Fenton reactions).^38,42^ Based on this model, when δ^13^C_sample_ yields −22‰, the conclusion is that the material is fully marine in origin. Similar mixing models, but with C/N ratios, have been also widely used^43^ and lead to the same conclusions.
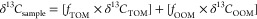
1Here, we propose an alternative explanation for the different characteristic δ^13^C and C/N values of organic matter in terrestrial and marine systems. This is based on the fact that fluvial processing removes massive amounts of carbon due to degradation (about 2.12 Pg-C of carbon, which is equivalent to 66% of the total riverine carbon inputs).^1−3^ Furthermore, given that the annual oceanic burial of organic matter is estimated to be 0.15 Pg-C,^6^ if that organic matter is entirely terrestrial (given that <1% of algal organic matter survives degradation),^16^ the total amount of outgassed carbon would be 2.75 Pg-C, equivalent to 95% land-to-ocean-sediment degradation. Degradation of TOM is largely driven by reactive intermediates, namely, reactive oxygen species (such as hydroxyl (•OH) and peroxyl (•OOH) radicals, singlet oxygen (^1^O_2_), superoxide (O_2_^•-^)) causing oxidation reactions. Reactive oxygen species are ubiquitous in soil, fluvial, and oxic sedimentary environments and are produced during photochemical irradiation;^44−46^ biomass decomposition;^47,48^ interactions with iron,^49^ manganese,^50,51^ or other metals (on particulate surfaces or in the dissolved phase); and even by enzymes — microbes commonly utilize extracellular enzymes to produce reactive oxygen species, which degrade larger molecules into smaller, more digestible substrates.^52−54^ Thus, environmental oxidation can be both biotic and abiotic. Furthermore, oxidation is not only a process occurring due to the presence of sunlight, as commonly perceived, but it can also occur in dark environments (groundwater channels or in the deep ocean), where TOM interacts with minerals or is degraded by microbes to turn it into digestible substrates. In turbid rivers, where photochemical and microbial pathways appear to play a minor role,^55^ Fenton reactions driven by suspended mineral material likely play a bigger role leading to high reactive oxygen species concentrations.^56^ Conversely, in oceanic systems, reactive oxygen species are commonly at very low steady-state concentrations in the water column,^57^ partially due to quenching by salts,^58^ but it is likely that TOM remains under a strong oxidation gradient in proximity to surfaces of sinking mineral particles and in oxic sediments of continental shelf/slopes, where the concentrations of such reactive species will be much higher.^59^

We have previously shown that upon oxidation, the molecular properties of terrestrial samples can become marine-like.^56,60−67^ More specifically, lignin, upon oxidation by reactive oxygen species, loses its characteristic features and becomes oxidized to aliphatic molecules,^61−63^ with a concurrent significant release of CO_2_. Some of the oxidation by-products appear to be refractory to further oxidation^56,68,69^ and thus might accumulate over millennia. Based on this, we hypothesize that as TOM is oxidized during its transport from land to the oceans, the undergoing molecular transformations releasing CO_2_ would result in a significant isotopic fractionation (i.e., the δ^13^C_TOM_ in eq 1 does not remain at a constant value). This oxidative fractionation would yield organic residues with δ^13^C_sample_ values that progressively resemble OOM in isotopic features. We test this hypothesis by performing *in vitro* oxidation studies of eight soils, aiming to represent diverse TOM materials. We employed a strong oxidation gradient in our experiments to achieve above 66% carbon loss (estimated to occur to TOM between land and estuaries/open ocean) and approach 95% carbon loss, which is estimated to occur to TOM between land and oceanic sediments (assuming minimal algal contributions).^16^

## 2 Materials and Methods

### 2.1 Samples

Bulk soil samples and initial organic matter contents prior to experimentation are given in Table 1.

- Hillsborough Soil: a riverbank soil of the Hillsborough River (Florida). The river arises from the Green Swamp, also known as the “Heart of the Floridan Aquifer”, and flows 60 miles to an outlet in the city of Tampa on Hillsborough Bay. Its watershed was formerly covered by a rich, old-growth forest of Bald Cypress, Longleaf Pine, and Sand Live Oak. After decades of logging, the watershed’s cover has shifted to recently grown Water Ash and Water Locust trees. The soil was collected on 02 April 2019 at coordinates 28° 05′ 16.8″ N and 82° 20′ 56.3″ W. After removal of large debris, the soil was homogenized, sieved, oven-dried at 105 °C, and stored in the dark to prevent degradation during storage.
- Santa Fe Soil: a riverbank soil of the Santa Fe River (Florida). The river arises from Lake Santa Fe in northern Florida and flows westward for 75 miles until it empties into the Suwannee River. The Santa Fe River is a slow-flowing river in a heavily forested watershed (mainly with Bald Cypress). The soil was collected on 03 April 2019 at coordinates 29° 55′ 22.8″ N and 82° 25′ 37.8″ W. After removal of large debris, the peat was homogenized, sieved, oven-dried at 105 °C, and stored in the dark to prevent degradation during storage.
- Elliott Soil: a standardized fertile prairie soil purchased from the International Humic Substances Society (IHSS; https://humic-substances.org/). This sample was obtained from an undisturbed area on the grounds of the Joliet Army Ammunition Plant near Joliet, Illinois, currently managed by the US Forest Service as the Midewin National Tallgrass Prairie Reserve. It is a poorly drained soil on moraines and till plains having loess or silty clay loam glacial till.
- Caitlin Soil: a deep, moderately well drained silt loam soil on a 2% till plain in a cultivated field in Ogle County, Illinois. It is a fine-silty, mixed, superactive, mesic Oxyaquic Argiudoll. The soil was sampled and standardized by the U.S. Department of Agriculture. The soil is formed in loess or other silty material and in the underlying loamy calcareous till (slope ranging from 0 to 15%).
- Sharpsburg Soil: a deep, moderately well-drained silty clay loam soil formed in loess in Lancaster County, Nebraska. It is a fine, smectitic, mesic Typic Argiudoll. The soil was sampled and standardized by the U.S. Department of Agriculture. This soil is formed on interfluve and hill slopes on uplands (slope ranging from 0 to 18%) and on treads and risers on stream terraces in river valleys.
- Pahokee Peat: a standardized peat soil purchased from the IHSS. It is an agricultural soil of the Florida Everglades obtained from the University of Florida Belle Glade Research Station. This is a poorly drained soil formed by organic deposits from freshwater marshes accumulating over limestone bedrock.
- Okefenokee Peat: a peat from the largest blackwater swamp in North America. This is a highly acidic (pH 3 to 4) low-nutrient environment.^70^ Its Spodosol soils are formed by the deposits of muck and peat mixed with fine salt and silt.
- Dismal Swamp Peat: a peat from a blackwater wetland in Virginia that has high iron loadings. Its hydric soils are formed under repeated saturation, long enough to develop anaerobic conditions in the upper horizons. The peat is formed by fast accumulation of carbon and slow decomposition yielding an organic-rich peat.^71^

**Table 1:** Bulk Geochemical Characteristics of the Initial Terrestrial Organic Matter (TOM) Materials^a^

**TOM**	**sampling location**	**TOC** (wt %)	**Fe** (wt %)	**Fe/TOC** (mmol/mol)
Hillsborough Soil	Thonotosassa, Florida	4.5	0.2	6.9
Santa Fe Soil	Gainesville, Florida	8.1	0.4	11.8
Elliott Soil	Joliet, Illinois	3.0	2.0	165.9
Caitlin Soil	Monroe Center, Illinois	2.6	2.4	195.9
Sharpsburg Soil	Sharpsburg, Iowa	1.6	2.6	360.5
Pahokee Peat	Belle Glade, Florida	46.5	1.7	7.9
Okefenokee Peat	Folkston, Georgia	49.3	0.2	1.0
Dismal Swamp Peat	Suffolk, Virginia	57.7	0.2	0.6

a TOC = total organic carbon (quantified as described below). Iron (Fe) was quantified as described previously.^56^

### 2.2 Oxidation

Oxidation of TOM samples was performed by suspending 2 g of dried material (105 °C for 12 h) in 10 mL of 1 M H_2_O_2_ (Fisher, 30%), with iron concentrations unperturbed from the native sample compositions (Table 1). Control experiments were done similarly but with ultrapure water (18.2 MΩ·cm). Experiments were performed in acid-cleaned and pre-combusted 20 mL glass vials, which were kept open to the air to ensure constant aeration and steady gas release, avoiding pressure buildup. As the eight soil samples were largely insoluble in water, once the aqueous H_2_O_2_ was added to the soils, acid-cleaned and pre-combusted rods were used to break any formed clumps and homogeneously distribute the peroxide solution. The formed suspensions were let react for 48 h at room temperature in a dark fume hood (vials were covered with a large KimWipe). Then, the peroxide solution was evaporated for 12 h at 105 °C to retain the particulate and dissolved components and also to quench the oxidation reactions for obtaining samples at exact time points.

Considering that the oxidation had continued to occur during sample drying, the total oxidation is estimated to be 60 h (2.5 days) per time point. After drying, a sample was sacrificed, and the remaining samples were used for further oxidation by adding new 10 mL of 1 M H_2_O_2_ for another 2.5 days of oxidation. This is graphically explained in Figure S1 in the Supporting Information (SI). Thus, oxidation was subsequently performed in six cycles for a total oxidation time of 15 days. Section 1 of the SI includes more details on the oxidation protocol, justifications for the choices of experimental conditions, and results from ancillary control experiments (Figure S2) and milder oxidation studies with 0.1 M H_2_O_2_ (Figure S3). In summary, the *in vitro* experimental conditions were chosen to ensure that the TOM samples experience a strong oxidation gradient that will yield significant carbon losses (minimum of 66% and above 90%). This would allow for testing if the carbon isotopic composition, C/N ratios, and bulk organic structure become altered at significant carbon losses comparative to carbon losses characteristic for TOM export from land to estuaries and the open ocean (∼66%) and to deep sediments (above 95%).

### 2.3 Elemental and Isotopic Characterization

Dried and homogenized samples were analyzed using a ThermoScientific Flash EA instrument equipped with a thermal conductivity detector and a Delta V Plus isotope-ratio mass spectrometer. The total organic carbon (TOC) content and isotopic composition (δ^13^C) were obtained on acid-digested samples (20% HCl to remove carbonates such as siderite^72^). Nitrogen content was obtained on unacidified samples. Samples were wrapped in tin capsules before combustion at 1010 °C. TOC and nitrogen values were obtained in triplicate (technical replicates), while δ^13^C values were measured three times on the same sample (instrumental replicates) and averaged into a single measurement. Carbon isotopic compositions were peak-size-corrected and scale-corrected using laboratory and authentic reference standards (glutamic acid: δ^13^C = −13.90‰; USGS40: δ^13^C = −26.39‰; USGS41a: δ^13^C = +36.55‰; and tyrosine: δ^13^C = −24.90‰). δ^13^C results are reported in delta notation relative to that of Vienna Pee Dee Belemnite.

The fraction of residual carbon (*C*_*t*_/*C*_0_) after each oxidation cycle was calculated using the TOC data accounting for carbon losses resultant from carbon remineralization (eq 2). This was done by keeping track of the mass changes of organic carbon present at each time point *t*. Carbon losses are determined as 1 – *C*_*t*_/*C*_0_.
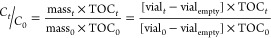
2

### 2.4 Isotope Modeling

Evaluating *C*_*t*_/*C*_0_ over the course of oxidation indicated that multiple components degraded simultaneously (Figure S4).^73^ Evaluating δ^13^C over the course of oxidation indicated that there is a significant isotopic fractionation (Figure S5) requiring the implementation of a kinetic isotopic effect (KIE) framework.^73^ The KIE is a constant describing how a given process (here, oxidation) changes the δ^13^C of organic matter as the reaction proceeds (eq 3). Note that the notation for δ^13^C is simplified to δ (δ_0_ is the starting δ, whereas δ(*t*) is δ at a certain time *t* of biogeochemical processing).
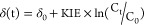
3

Considering these results, an isotope mass balance model^74^ was developed to interpret the changes in δ^13^C relative to carbon losses (eq 4).
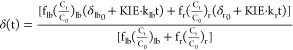
4

This model applies the basic principle of isotope mass balance and assumes that the initial bulk organic carbon (mass, with associated isotope ratio) is quantitatively either converted to an oxidized product (in our model, assumed to be the labile TOM fraction *f*_lb_ turning into CO_2_ or other volatile molecules) or left behind as residual refractory material (refractory fraction *f*_r_) that accumulates from *f*_lb_. These two components have rate constants *k*_lb_ and *k*_r_, respectively. The carbon loss in this study was due to the oxidation of organic matter to CO_2_ or other gases as well as the evaporation of volatile species during sample drying. The final model (eq 4) is able to generate best-fit values for *f*_lb,_ the KIE, and the starting δ^13^C value of the labile and refractory fractions (labeled δ_lb0_ and δ_r0_, respectively). A key feature of this model is that it assumes that the reactions are unidirectional (i.e., oxidation is irreversible leading to carbon losses, with gases, such as CO_2_, leaving the system).

Considering the number of unknowns, conservative (wide boundary) constraints were placed to yield realistic results. The value of *k*_lb_ is calculated from the initial slope of ln(*C*_*t*_/*C*_0_). The value of *k*_r_ is assumed to be ≤ the slope of the late time points of ln(*C*_*t*_/*C*_0_) (Figure S4). δ_lb0_ is assumed to be ≥−33‰ (a reasonable minimum threshold for C_3_ plant biomass), and δ_r0_ is constrained to be ≥ the bulk δ^13^C value at the slope break between *k*_lb_ and *k*_r_ but ≤5‰ more positive than the highest measured bulk δ^13^C value. The KIE was allowed to be between 1 and 4‰. Because *f*_lb_ + *f*_r_ = 1, it is necessary to only fit *f*_lb_.

The final model was fitted to δ^13^C, *C_t_*/*C*_0_, and *t* data by implementing a simulated annealing routine (simulannealbnd function) of the MATLAB Global Optimization Toolbox (MATLAB V2023a) using Monte Carlo resampling approaches (2000 runs per trial). This approach minimizes the error cost function for multivariable optimization problems within prescribed bounds and is suitable for both linear and nonlinear problems. The development of the model, its constraints, and underlying assumptions are described in detail in Section 2 of the SI. Model results and associated uncertainty ranges are shown in Table S1. It must be noted that the rate constants determined in this study are not representative of environmental degradation rates, as the time scale employed here is arbitrary (laboratory oxidation). These values should only be used for comparing rate constants among samples within this study and are not suitable to incorporate into global carbon cycle models.

### 2.5 Structural Characterization by ^13^C Solid-State NMR

Dried powdered samples (before acid digestion) were packed in a 4 mm zirconia (ZrO_2_) rotor with a polychlorotrifluoroethylene (Kel-F) cap. Analysis was done on a 400 MHz (9.4 T) Bruker BioSpin AVANCE II spectrometer fitted with a 4 mm magic angle spinning probe at the College of Sciences Major Instrumentation Cluster (COSMIC) facility at Old Dominion University (Norfolk, VA). One-dimensional ^13^C NMR spectra were acquired using the nearly quantitative multipulse cross-polarization (multiCP) pulse program.^75^ Samples were spun at the magic angle at 14 kHz and analyzed using a relaxation delay of 1 s, 3000 scans, five cross-polarization segments, and a total contact time of 3.30 ms. The obtained spectra were phased, calibrated to an external glycine standard, and multiplied by an exponential window function (EM) of 200 Hz. Spectra were then baseline-corrected and integrated in the following ranges: 0–45 ppm (methyl and methylene); 45–60 ppm (αC in peptides); 60–95 (O-alkyl); 95–110 ppm (anomeric C), 110–145 ppm (aryl), 145–165 ppm (aryl-O), and 165–215 ppm (CO). All data were processed using the Bruker TopSpin 4.0.7 software. The obtained integrals were then used in a molecular mixing model^76^ to determine the approximate carbohydrate-like, protein-like, lignin-like, lipid-like, and char-like contributions.

## 3 Results and Discussion

### 3.1 Oxidation-Driven δ^13^C Fractionation toward an Oceanic Isotopic Signature

**Figure 1:**
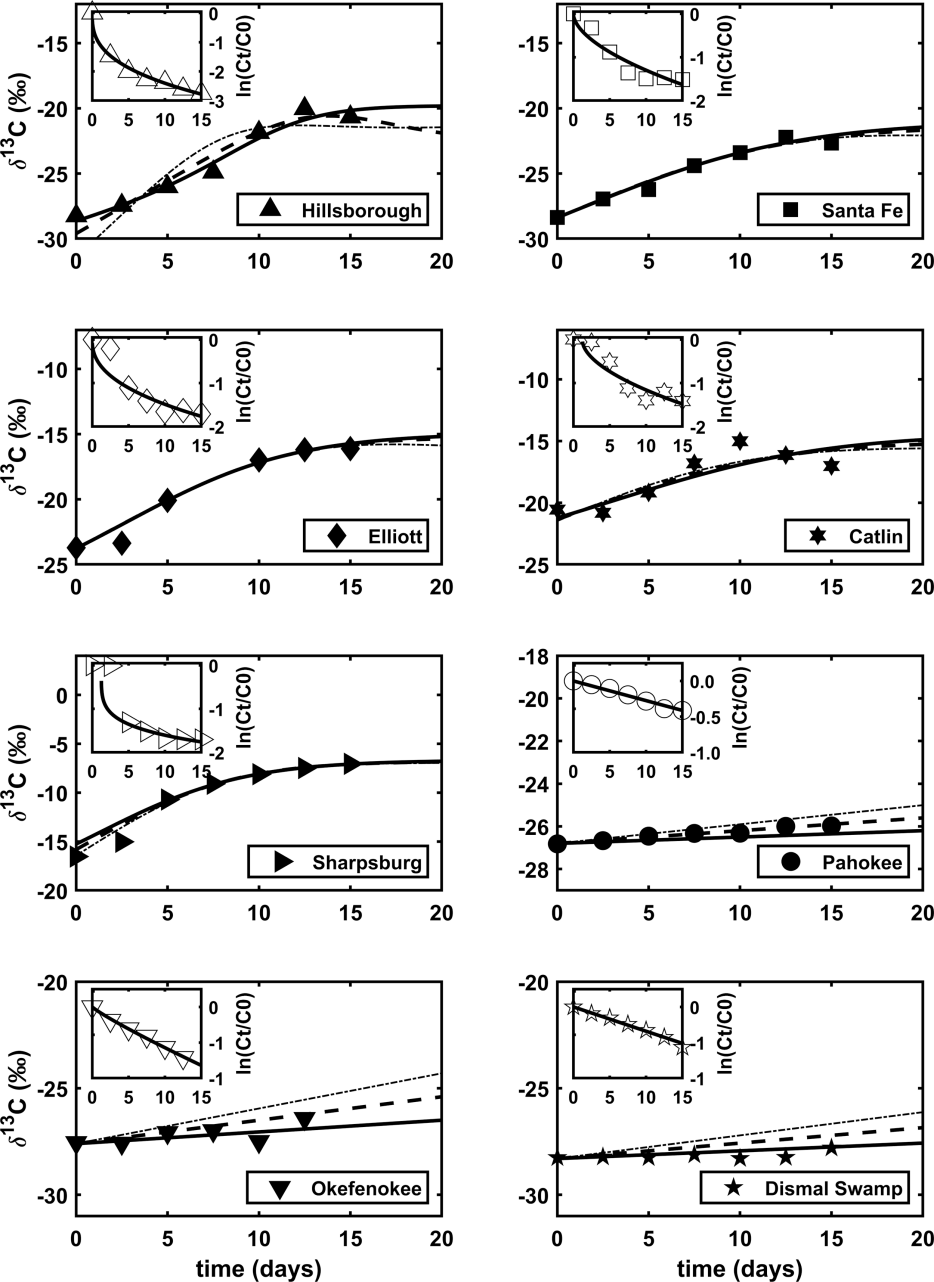
Measured δ^13^C values and the remaining carbon after oxidation (inset panels; see also Figure S4) for the eight soil samples. Isotope data are fit with a two-endmember mixing model containing labile and refractory fractions. Solid, long-dash, and dash-dot lines in the isotope panels represent kinetic isotope effect (KIE) values of 1, 2, and 3‰, respectively; goodness of fit is insignificantly different for KIE = 1 and 2‰ but deteriorates at ≥3‰. Error bars are not shown for the sake of clarity.

Bulk material from eight terrestrial systems was subjected to oxidation to quantify changes in the ^13^C isotopic signatures. Five of the eight samples experienced carbon losses up to 94% (Figure 1 and Figure S4), which yielded concomitant large ^13^C enrichments (4 to 9 ‰) with increasing oxidation time (Figure 1 and Figure S5). This contrasts previous studies testing the effect of oxidation on δ^13^C values,^42,77^ which had only tested δ^13^C at lower carbon losses (below 50%). The initial values of δ^13^C for Hillsborough and Santa Fe are characteristic of TOM derived from C_3_ plants (−28.3 and −28.4‰, respectively). The final values for these soils are ^13^C-enriched (−20.7 and −22.7‰, respectively), terminating within the range of δ^13^C values commonly interpreted as characteristic of phytoplankton-derived OOM.^32^ These samples lost 94 and 78% of their carbon after oxidation, respectively, finishing with 2.0 and 0.3% TOC contents, respectively, which TOC values are typical of marine sediments. While the Pahokee, Okefenokee, and Dismal Swamp peats also had similar initial values of δ^13^C (−26.8, −27.6, and −28.3‰, respectively), their higher initial TOC contents (46, 51, and 59%, respectively) and correspondingly smaller fractional loss of material (34, 51, and 43%, respectively) yielded smaller shifts in their δ^13^C values (to −26.0, −26.4, and −27.8‰, respectively). All five of these samples are from semitropical, wet environments with low Fe/TOC ratios of 0.6–12 mmol/mol (Table 1).

The Elliott, Caitlin, and Sharpsburg soils are of much higher Fe/TOC ratios (166, 196, and 360 mmol/mol, respectively) and have initial values of δ^13^C characteristic for systems of mixed C_3_ and C_4_ inputs (−23.7, −20.6, and −16.5‰, respectively). Despite Elliott Soil’s proximity to agricultural regions, it is unlikely that its initial δ^13^C value represents a significant input of C_4_-derived agricultural carbon (from corn), because the Midewin prairie reserve was established on the grounds of a former military munition plant that had not been in recent cultivation. Instead, this soil likely contains inputs from native C_4_ grass species (e.g., *Andropogon spp.*).^78^ The Elliott Soil showed a similar behavior to the Hillsborough and Santa Fe samples, losing 82% of its TOM to oxidation in tandem with a 7.6‰ increase in its δ^13^C value. The Caitlin and Sharpsburg soils likely do contain initial C_4_ carbon derived from corn, as both of these soils are from agricultural areas.^79^ These samples lost 76 and 82% of their carbon, respectively, after oxidation, finishing with δ^13^C values of −17 and −7‰, respectively. The fact that such high δ^13^C values (e.g., −7‰) are not commonly observed in environmental matrices indicates that there is significant mixing of oxidized material with fresh material (or with materials of different origins), obscuring the contributions of highly ^13^C-enriched inputs, and highlights the necessity of muti-component mixing models in this line of research.

Despite the higher Fe/TOC ratios of all three temperate soils, their ^13^C enrichment (4 to 9‰) was similar to that of the tropical soils with much lower Fe/TOC, suggesting similar oxidative degradability across different iron levels. This is surprising, as increasing iron proportions to carbon should yield stronger oxidation.^59^ Quantitatively, there were very poor and insignificant correlations between the isotopic enrichment and Fe/TOC ratios (Pearson *r* = −0.63, *R*^2^ = 0.40, *p* = 0.09; Figure S7A) and between the total carbon loss and Fe/TOC ratios (Pearson *r* = −0.46, *R*^2^ = 0.21, *p* = 0.26; Figure S7B). This is likely due to the fact that our iron measurements (Table 1) represent total iron, which is comprised of seven forms: (1) carbonate-associated iron (e.g., siderite and ankerite), (2) easily reducible oxides (e.g., ferrihydrite and lepidocrocite), (3) reducible oxides (e.g., goethite, hematite, and akageneite), (4) magnetite, (5) poorly reactive sheet silicate iron, (6) pyrite, and (7) unreactive silicate iron.^80^ These different iron phases have different reactivity, and their ability for reductive dissolution ranges from hours to beyond 10,000 years.^81^ Thus, iron-poor samples having highly reactive iron forms would be labile to oxidation, which is likely the case for the two riverbank soils (Hillsborough and Santa Fe). Conversely, it is possible that iron-rich soils having unreactive iron forms might be refractory to oxidation, although such samples appear to not be present in our current dataset (Figure S7).

To further explore the fractionation of δ^13^C by oxidative processing, we employed kinetic modeling (eq 4) hypothesizing that the bulk samples have multiple underlying components with different degradation rate constants. These components collectively result in a decreasing loss rate and plateau-like behavior of the evolving δ^13^C values. Plots of relative carbon content show best fit to a power law, *k*(*t*) = *k*_max_*t*^b^, consistent with the “multi-g” or “multi-k” framework commonly applied to marine sediments (inserts of Figure 1; Figure S4).^82,83^ However, while a time-dependent change in *k* effectively explains the *C*_*t*_/*C*_0_ profiles, the isotope data are inconsistent with a homogeneously accumulating pool of isotopically and molecularly uniform, residual TOM (Figure S5). Oxidation of a single substrate in a closed-system oxidation reaction would yield a Rayleigh-type exponential increase in δ^13^C values of the remaining unoxidized material assuming a constant kinetic isotope effect (KIE) of oxidation. Instead, the pattern of δ^13^C values implies the mixing of non-contemporaneous and/or molecularly heterogeneous carbon pools, suggesting that the TOM can be divided into labile and recalcitrant fractions (*f*_lb_ and *f*_r_, respectively). The *f*_r_ is assumed to originate from the transformation of labile material during oxidation and/or to include residues that accumulated *in situ* during soil formation. Thus, in aggregate, *f*_r_ may not be of concurrent age to the *f*_lb_. This framework is consistent with other analytical methods such as radiocarbon dating,^84^ which also commonly assess TOM using similar two-component modeling approaches.

The modeling reveals that in the Hillsborough Soil, the labile component comprises ≥98% of the total carbon, with a loss rate constant of 0.40 day^–1^ (Table S1). Note that the units of these rate constants (day^–1^) are for the employed oxidation conditions and correspond to laboratory oxidation time; thus, their values are not comparable to environmental oxidation rates. The minor, refractory component appears to have a δ_r0_ value of ca. −20 to −24‰ and a loss rate constant of <0.004 day^–1^. The model solutions for the Santa Fe Soil are similar, with a δ_r0_ value of ca. −21‰ and a loss rate constant of <0.004 day^–1^, but with a smaller predicted initial contribution of labile material (*f*_lb_ = 0.65–0.73). The labile fraction in Elliott Soil is in a similar proportion (*f*_lb_ = 0.72–0.81), but the isotopic endmember value predicted for the refractory fraction approximates that of C_4_ plants (ca. −15‰). For the Elliott and Caitlin samples, in addition to incorporating C_4_-derived plant material, the high Fe/C ratios (166 and 196 mmol/mol) may promote oxidative loss and ^13^C fractionation of the TOM as it forms. This appears to be the case also for the Sharpsburg soil, in which the refractory component is estimated to have a signature of ca. −7‰ and has the lowest amount of estimated labile component (*f*_lb_ = 0.65–0.69). This indicates that the Sharpsburg soil may have already been significantly oxidized prior to sampling as well as contain significant C_4_ plant inputs. Finally, the Pahokee, Okefenokee, and Dismal Swamp peats experienced too little loss of material to model their solutions for two different carbon fractions; rather, all carbon was assumed to be in the *f*_lb_ pool with a single rate constant *k*_lb_ (ranging from 0.03 to 0.06 day^–1^).

Lastly, the modeling revealed that the optimal KIE for oxidation ranges from 1 to 2‰. This KIE is indistinguishable from the magnitude of carbon isotope enrichment generally observed for oxic water columns and sediments^86^ and for the aerobic microorganisms characteristic of these degradation conditions.^87^ This suggests that the results obtained from laboratory experimentation in this study are comparable to fieldwork-based findings even though we used strong laboratory oxidation conditions.

### 3.2 TOM Oxidation Yields an Oceanic-like Aliphatic- and Nitrogen-rich Residue

**Figure 2:**
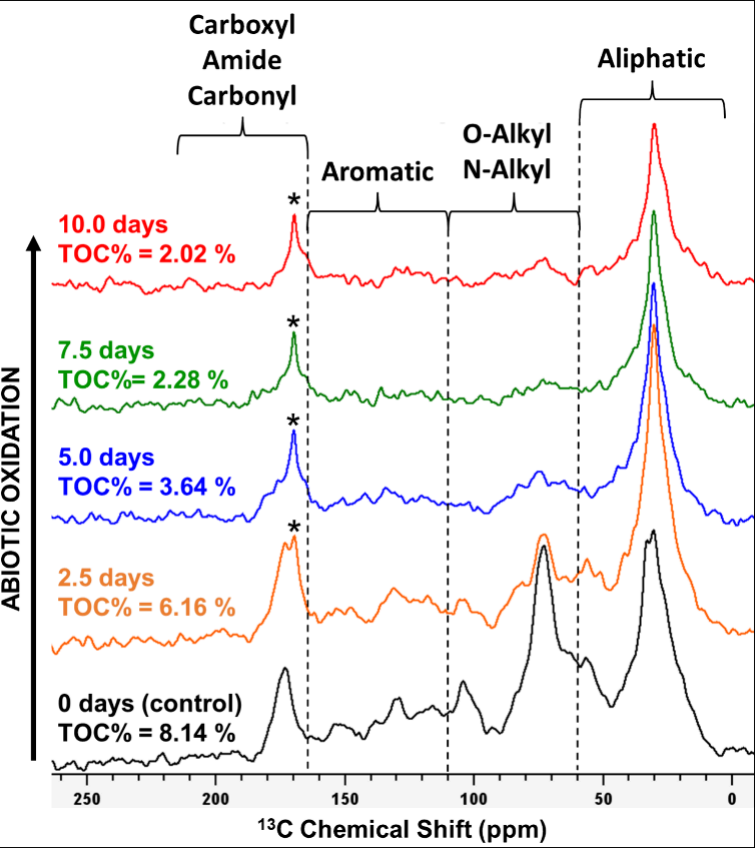
Solid-state ^13^C NMR spectra of Santa Fe Soil showing the loss of total organic carbon (TOC) with increasing oxidation. Aromatic (mainly from lignin), O-alkyl and N-alkyl (mainly from carbohydrates and proteins), and carboxyl signals diminish, whereas the oxidized residue became gradually enriched in aliphatic remains as well as oxalate (labeled with an asterisk).

Solid-state ^13^C NMR analysis revealed significant changes in organic structures upon oxidation of soil TOM (Figure 2 and Figures S8–S10). NMR has a wide analytical window capable of characterizing nearly all carbon-containing structures, and it can be almost fully quantitative, permitting estimates of different classes of functional groups.^88^ Unfortunately, this technique is affected by minerals (e.g., sand) and paramagnetic species such as iron. Thus, samples containing large amounts of iron-bearing minerals are nearly impossible to characterize, and thus, obtaining numeric NMR data in this study has been limited to the samples that yielded spectra of acceptable quality (Santa Fe Soil and Pahokee Peat, Figure 2 and Figure S8). The other samples (Hillsborough and Elliott soils) yielded NMR spectra with low signal-to-noise ratios due to their mineral-rich matrices (Figures S9 and S10), and estimates of functional group contributions could not be obtained. The remaining soils were not analyzed either due to their even higher iron contents or due to their small carbon losses.

Untreated initial samples of Santa Fe Soil and Pahokee Peat showed fractional group areas of 31 and 25% aliphatic (0–45 ppm), 43 and 35% O-alkyl and N-alkyl (45–110 ppm), 17 and 27 % aromatic (110–165 ppm), and 9 and 13 % carboxyl, amide, and carbonyl (165–215 ppm) functionalities (Figure 2 and Figure S8, respectively), a composition that is broadly characteristic of TOM.^18^ The emergent sharp peak at 169.4 ppm in the oxidized samples represents the accumulation of what appears to be hydrogenoxalate (Figure S11). Oxalate (C_2_O_4_^2–^) and its monovalent form detected here (HC_2_O_4_^–^) are likely products from the liberation of radical carboxyl functionalities (•COOH), which combine, and the formed oxalates likely bind with metals present in the soil (e.g., Ca^2+^, Mg^2+^, Al^3+^, and Fe^2+/3+^). Oxalate may also form from the oxidation of other moieties (e.g., ring-opening reactions of phenols^61^ or larger aromatics^66^ leading to muconic-acid-like compounds). This shows that oxalate, while generally viewed to be of biotic origin,^89^ is also a product of abiotic oxidation reactions as previously observed during the oxidation of coal.^90^

Upon oxidation, the ^13^C NMR spectra progressively lose peak intensities in chemical shift regions above 45 ppm, particularly in the O-alkyl and N-alkyl and the aromatic regions (i.e., 45–160 ppm). Partial loss of carboxyl/amide functional groups is also observed. Residual signals, thus, are strongly aliphatic, with final profiles having 30–55% of their total spectral intensity <45 ppm. The loss of aromatic signals during oxidation is likely due to the oxidative reactivity of lignin.^61^ After oxidation, the aromatic content of the degraded TOM resembles levels commonly observed for marine sediments, as does the overall profile of the ^13^C NMR spectra (Figures 2 and S8).^18,91^ The observed structural changes are associated with a significant loss of carbon in both samples (Figure 1).

The solid-state ^13^C NMR spectra were subjected to deconvolution into biopolymeric components^76,88^ as done previously for testing the hypothesized mixing of TOM and OOM.^18^ Here, we calculated the change in carbon associated with lignin, carbohydrates, proteins, lipids, and char as a function of oxidation time (description in Section 4 of the SI). The data for Santa Fe Soil follow exponential functions with *R*^2^ values >0.92 for all components except lipids (Figure S12). Within these trends, the lignin and lipid (polymethylenic) components show incomplete losses, while carbohydrates, proteins, and char biopolymers were completely degraded. The exponential decrease in polymethylenic components and their imperfect fit (*R*^2^ = 0.84) are explained by the simultaneous degradation of lipids and their oxidative production from lignin or other aromatics.^61,66^ Similar trends have been observed for sinking coastal particulate matter in the Saanlich Inlet of the Pacific Northwestern United States,^92^ as assessed by the deconvolution of specific molecular biomarkers representing each biopolymer class.

For the Pahokee Peat, the carbohydrate, protein, and char components decreased with oxidation time, but the polymethylenic and lignin biopolymers remained stable (Figure S13). Polymethylenic compounds are inherently most resistant to oxidation, whereas biopolymers like carbohydrates and proteins are labile and readily oxidized. These differences in behavior relative to Santa Fe Soil reflect the limited fractional extent of degradation for the Pahokee experiment (a loss of only 34% of the carbon inventory compared to 78% loss for Santa Fe; Tables S3 and S7). The inertness of the Pahokee, Okefenokee, and Great Dismal Swamp peats is likely due to multiple factors: (1) the high initial organic content of the peats, (2) the insufficient accessibility of H_2_O_2_ and the produced radicals to reach reactive sites in the organic-rich peat, or (3) the insufficient quantity of H_2_O_2_ used for the reaction conditions (i.e., due to the high amounts of organic matter, the reaction could be H_2_O_2_-limited). Also, a noticeable hydrophobic mixing behavior was noted for these samples when suspended in hydrogen peroxide, suggesting inefficient mixing and accessibility of H_2_O_2_ to reactive TOM sites.

**Figure 3:**
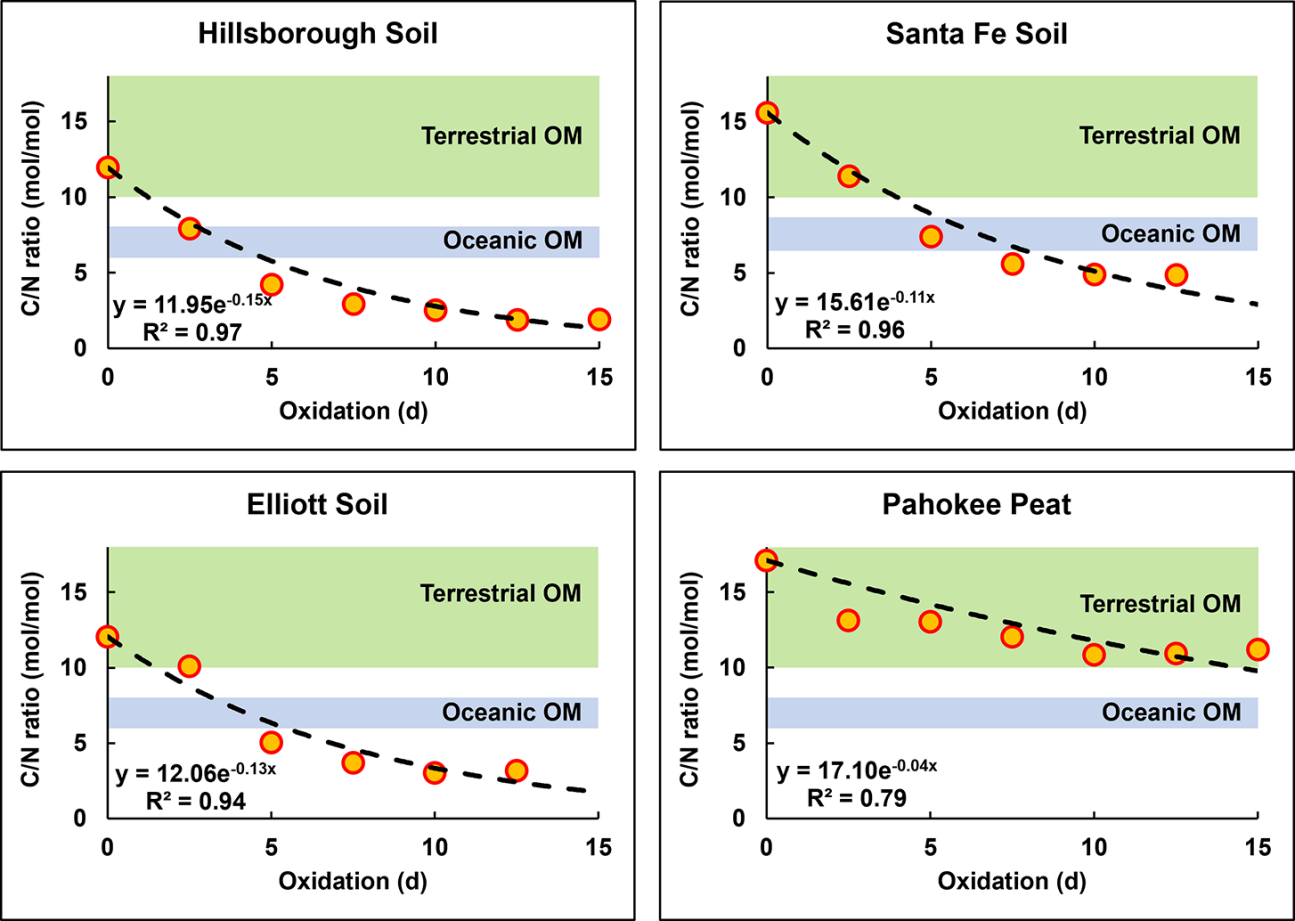
C/N ratios determined from elemental analysis of control and oxidized soil samples. Data are fitted to exponential equations (*y* = *ae*^–*bx*^) and are contextualized to C/N ranges typical for terrestrial organic matter (C/N above 10, green area) and oceanic organic matter (C/N ranging 6–8, blue area). The intercept parameter “*a*” displays the starting C/N ratio of each soil. Error bars are not shown for clarity.

Another common differentiator between TOM and OOM has been the molar C/N ratio. TOM typically has C/N ranging 10–15, but it can be higher as lignin has C/N ≈ 50. OOM, on the other hand, typically has C/N ranging 6–8, which is close to the C/N of proteins (C/N ≈ 4).^33^ C/N ratios, similarly to δ^13^C measurements, have been widely used for modeling TOM and OOM fluxes and reservoirs.^43^ Results from oxidation experiments of four select samples show that the C/N ratio of TOM exponentially decreases from >10 down to ≈2 during oxidation (Figure 3). Thus, TOM likely does not retain a constant C/N ratio during its export from land to the ocean, complicating the traditional use of C/N ratios for differentiating TOM and OOM (using models like eq 1). The observation of unusual C/N values for environmental matrices (2–5 mol/mol) indicates that significant mixing occurs in environmental systems where highly oxidized TOM with low C/N is mixed with other materials of higher C/N (e.g., fresh TOM) to yield a mixture of purely terrestrial origin that mimics OOM C/N signatures.

## 4 Environmental Implications

Subjecting TOM to oxidation alters its chemistry and δ^13^C composition to the point that the properties of its residual material resemble those commonly classified as characteristic of marine-derived organic matter. Consequently, it is difficult to argue that the bulk, macromolecular properties of OOM are unique, either structurally or isotopically, or that they can be differentiated from the oxidation by-products of TOM. Instead, our work here suggests that a degradation-resistant fraction of TOM (i.e., *f*_r_) persists and accumulates organic functional group transformations and ^13^C enrichment sufficient to imply that “camouflaged TOM” could represent a major, if not majority, fraction of oceanic dissolved, particulate, or sedimentary OOM. Majority preservation (i.e., >0.075 Pg-C) of TOM would require 15–20% of the delivered 0.48 Pg-C of TOM to be sufficiently recalcitrant to escape oxidation, similar to the chemical resistance observed here for the Santa Fe, Elliott, Caitlin, and Sharpsburg samples (*C_t_*/*C*_0_ of 18–24% at the final experimental time points) and for prior estimates of TOM burial efficiency in deltas and continental margins.^93^

The disappearance of lignin phenol biomarkers has been used to suggest complete mineralization during the transfer of TOM from land to coastal waters^94^ and is a main criterion supporting the idea that TOM is a minor contributor to OOM. However, the inefficiency of removal processes (e.g., photochemical mineralization and bacterial respiration) in rivers and estuaries indicates that these removal processes are not sufficient to allow for the complete loss of lignin.^95−97^ One possibility is that lignin undergoes chemical transformations, as shown in Figure 2. We have shown that such alterations of lignin occur through both biotic and abiotic oxidative processing without complete mineralization.^61−65^ Exposures to reactive oxygen species from enzymes,^53^ photochemistry,^44−46^ or mineral–TOM interactions^49−51^ induce structural modifications such as loss of methoxy groups and ring hydroxylation followed by ring rupture. Thus, the remaining carbon atoms associated with lignin become unrecognizable structures and are undetected by the conventional analytical method of lignin phenol biomarkers, leading to massive underestimations of the contribution of TOM to the global ocean. Previously, we have shown that lignin can transform into carboxyl-containing aliphatic molecules (CCAM) commonly found in soils, peats, and swamps.^60,61,69,98,99^ Thus, TOM oxidation products contain aliphatic compounds, many of which are also more commonly known as carboxyl-rich alicyclic molecules (CRAM),^85^ generally assigned to OOM. In this study we also show that the nitrogen distribution in TOM (i.e., C/N ratios) is also severely impacted, and nitrogen appears to be concentrated in the residual structures.

The major implications of our findings are globally transformative considering that most global carbon cycle models treat terrestrial and oceanic systems as two separate pools of organic matter separated by a distinct boundary—i.e., with minimal cross-boundary transfer at the coastal margin.^8,10,93,100^ Instead, our data indicate that there likely is a major continuity between these pools, and the remineralization of TOM, as it enters the ocean margin, is better represented as a high-efficiency batch reactor^9^ having variable reaction rates for different molecular classes and dependent on oxygen exposure.^101^ This reactor then discharges an isotopically and molecularly transformed TOM residue into the marine environment. Our oxidation experiments mimic an accelerated version of these batch-loss dynamics and show that bulk δ^13^C values of −22 to −20‰ are easily achieved at residual TOC contents of 0.5–2.0%, i.e., typical organic loadings of buried marine sediments. This implies that TOM flows through a bottleneck that imprints a Rayleigh-like distillation onto the δ^13^C signatures of the remaining residues. The batch-reactor-like behavior is consistent with the vigorous degradation of large amounts of TOM (≈66%) prior to export at the mouth of rivers.^1–3^ Further oxidation of this TOM in the coastal margin to render 75–95% loss,^16^ leaving behind a refractory residual that is 4–9‰ enriched in ^13^C (assuming the KIE = 2‰), is realistic in the context of existing carbon cycling models. Overall, these results indicate that the entrainment of TOM into the oceanic compartments of the global carbon cycle must be reconsidered and re-evaluated in the context of oxidation gradients and organic matter burial rates existing along the land–ocean continuum. A corollary would be that the cycling of organic carbon derived from marine primary production may be so complete in oxygenated water columns that its contribution to sedimentary OM may be small in comparison to that of TOM.

While having crucial implications for current carbon cycling models, there are numerous other implications of a major role for the oxidative transformations of organic matter:

- Production of reactive intermediates, primarily reactive oxygen species, through Fenton, photochemical, or biotic pathways is expected to proceed at will in the presence of dissolved O_2_ or of available iron or other metals, in the presence of microbes, and in sunlit environments. These pathways are independent of each other; i.e., minerals will produce reactive oxygen species through Fenton reactions independently of biological activity or enzyme complement (either extra- or intracellular). Therefore, degradation of labile organic material by reactive oxygen species is globally widespread whenever water columns and porewaters are oxygenated and the organic matter contacts mineral-rich detrital material (or in the presence of sunlight).
- A major role for abiotic processes implies that traditional models of sediment biodegradation by enzymatic attack (typically by large enzyme molecules)^102^ and the resulting stability of pore-occluded or sorbed materials^103,104^ would underestimate the vulnerability of the pore-occluded or sorbed material to oxidative degradation unless it is fully encapsulated in protective matrices (e.g., within biominerals, foraminiferal calcite, diatom frustules, and/or coccoliths).^105^ Organic matter templates within biominerals may therefore be the only “native” OM that remains unaffected by oxidative fractionation and, thus, remain a good geologic archive. However, it would be important to consider that reactive oxygen species are small species that may access mineral-encapsulated sites and diminish the effectiveness of this encapsulation.
- For free and mineral-associated organic matter in aquatic systems, the lability of the type of biopolymer and the selective preservation of refractory polymers, combined with the generation of newly refractory geopolymers,^20,106^ may determine the fate, molecular composition, and terminal carbon isotope ratio of buried organic matter, ultimately governed by the tendency of the system toward oxidative exposure.^101,107^
- The interplay between abiotic and biotic degradation also has implications for the preservation of organic matter in anoxic environments because oxygen-free radical formation is suppressed in such systems. Anoxia—or a shorter oxygen exposure time—would be predicted to preserve residual TOM with a ^13^C-depleted composition closer to its original endmember value. Instances of ^13^C-depleted organic matter in near-shore restricted basins (e.g., Santa Barbara Basin,^108^ Santa Monica Basin,^109^ and Congo Canyon^110^) are commonly ascribed to inputs of microbial biomass from organisms expressing alternative metabolisms (i.e., associated with CH_4_ or with autotrophic consumption of ^13^C-depleted porewater dissolved inorganic carbon). Despite being quantitatively important sediment redox processes, it has been mysterious how such low-energy reactions could generate enough biomass to overprint the burial of C-rich bulk organic matter. By our model, 50% oxidative degradation of TOM before burial would consume 46–48% of the aromatic functionality and yield a bulk δ^13^C value of ca. −26 to −27‰ (from a starting value of −28‰; based on a KIE of 1 to 2‰), thus demonstrating that a lignin-like molecular character may be difficult to detect despite the origin of most of the sediment from degraded terrestrial material. Oxidation after burial is also possible if redox fluctuations (oxic/anoxic) exist between the sediment and the bottom layer (during redox fluctuations between the abyssal zone (in oceans) or hypolimnion layers (in lakes).^111^ Another possibility for anoxic sediments is that other oxygen-free reactive intermediates such as elemental sulfur (•S_n_), mercapto (HS•), or thiyl (RS•) radicals could cause analogous degradation reactions leading to similar elemental, molecular, and isotopic camouflaging.

In conclusion, the main takeaway of this work is that the oxidation of TOM has a significant impact on its elemental (C/N), structural (aliphatic vs aromatic character), and isotopic (δ^13^C) signatures leading to a marine-like ^13^C enrichment in the residual TOM, concomitant with an increase in aliphatics and a decrease in C/N ratios. These results are most pronounced when the oxidation is vigorous enough to remove >75% of the carbon (Figure 1). This is comparable to the expected carbon losses of TOM (66–95%) as it travels from land through estuaries and is potentially buried in marine sediments (assuming that these losses are from biotic and/or abiotic oxidation leading to quantitative outgassing). Although strong oxidation and high carbon losses may lead to extremes in C/N ratios (ranging 2–5), unusually high δ^13^C values (e.g., −7‰), and nearly pure aliphatic residues (Figure 2), mixing of such oxidized material with fresh material could easily result in OOM-like mixtures, as explained by our mixing model (eq 4). Thus, our results indicate that without independent knowledge of the extent of OM exposure to oxidative degradation, the source of dissolved, particulate, and sedimentary OOM cannot be determined solely from its elemental, macromolecular, or isotopic composition. Our findings imply that a significant fraction of OOM could feasibly originate from TOM, with critical implications for understanding sediment burial and global carbon budgets, and that the extent of oxidative degradation before burial could be a primary control on the final δ^13^C signatures of buried organic matter. These ideas have critical implications for understanding modern and ancient global carbon budgets. Thus, in future applications, the use of conventional lignin phenol, C/N, and δ^13^C mixing-model methods should be amended to account for the proposed oxidative camouflaging of TOM. Alternatives include implementing multi-component δ^13^C mixing models (e.g., eq 4), incorporating multiple isotope systems (^2^H/^1^H and ^14^C),^112^ and/or ultrahigh resolution mass spectrometry, the latter having shown promise in better constraining terrestrial and oceanic endmembers and showing survival of 50–76% of Amazonian TOM in the coastal ocean.^55^

## 5 Emerging Directions for Future Research

The findings of this work have the potential to cause a paradigm shift in organic biogeochemistry. Highlighted below are several trajectories for future research that need to be pursued to fully characterize the process of TOM camouflaging by oxidative degradation.

1. **Scaling to global rates:** A major challenge with the present work is that the quantified degradation rate constants of refractory and labile components (*k*_lb_ and *k*_r_) are in arbitrary day^–1^ units. Future research should determine a scaling factor to convert these rates to environmental scales. This can be done by paired laboratory–fieldwork studies in which the same TOM materials are oxidized in laboratory settings and, in parallel, TOM samples from the same environment are collected ensuring that they have experienced a natural oxidation gradient. Lastly, to ensure that TOM camouflaging occurs on global scales, samples from major terrestrial systems (e.g., Amazon, Congo, Mississippi basins) should be evaluated for their oxidative degradability and potential to become marine-like.
2. **More representative experimental methodology:** Future studies should consider refining the analytical protocol (Section 1 of the SI) to include higher-resolution time points (to provide more robust model solutions), include a control sample that has undergone wetting and drying with H_2_O for the same total number of cycles used, and perform replicate experiments for a better uncertainty analysis (including experimental and technical replicates). The heating step for reaction quenching should be also substituted with freeze-drying. Additionally, in natural biogeochemical conditions, dissolved species are constantly removed while particulate TOM is oxidized, whereas in our experiments, both the particulate and dissolved fractions were preserved. Thus, a flow-cell experimental design for oxidizing TOM should be considered. Lastly, TOM experiences biotic degradation, mixing with salts from seawater, diffusion, which leads to dilution, and overall transport downstream. Thus, TOM oxidation should be also researched in the context of such parallel physical and biogeochemical processes.
3. **Inorganic chemistry characterization:** Given that there were poor correlations between Fe/TOC ratios and oxidation results (total carbon loss, isotopic enrichment; Figure S7), it was suspected that not all phases of iron were involved in the reaction with H_2_O_2_. Future studies should involve sequential extraction and quantification procedures to quantify the seven phases of iron (iron oxides, magnetite, pyrite, etc.)^80^ as well as X-ray diffraction on whole TOM samples^113^ to assess if other minerals (clay, sand, etc.) are at play. Hypothesis-based experiments of reacting individual minerals with pure compounds or with mineral-free biomass should be also considered.
4. **Radical species and OM oxidation mechanism:** Given that the reaction of H_2_O_2_ with metals can yield a diversity of radical intermediate species (hydroxyl/peroxyl radicals, superoxide, etc.) and that they all vary in oxidative potential, it will be important to understand how each participates in the oxidation of TOM. Molecular probe methods or electron paramagnetic resonance can be used to determine radical speciation and their concentrations. Hypothesis-driven time-series experiments of individual radicals with individual compounds (e.g., lignin phenols with hydroxyl radicals) can be useful in determining the exact mechanism of reactions (following monitoring with ultrahigh resolution mass spectrometry, NMR, etc.). Furthermore, elemental and isotopic measurements of such experimental systems would allow for strengthening the assumptions and constraints used in modeling carbon degradation and isotopic fractionation (eq 4)
5. **Organic composition exploration:** Considering the observed differences in labile and refractory fractional composition of five of the samples (Table S1), it will be important to investigate what structural fractions these correspond to. We have done some preliminary work on this via solid-state ^13^C NMR (Figures S12 and S13), but future studies should more robustly investigate the composition of TOM undergoing oxidation (e.g., quantify the different concentrations of lipids, lignin, and condensed aromatics, which all vary in degradability) via the employment of HF demineralization protocols^114^ and more sensitive solid-state NMR methods.^115^ Nitrogen speciation methods, such as N K-edge XANES, should be also considered. It is also possible that the labile and refractory fractions of TOM (i.e., *f*_lb_ and *f*_r_) overlap with the dissolved and particulate operational fractions of TOM, which two pools cycle very differently, with the particulate fraction interacting with storage sediment pools in inland waters and coastal environments that introduce it to different oxidative gradients and transit times compared to the dissolved fraction. However, it is unlikely that the labile component *f*_lb_ is fully equivalent to the dissolved TOM fraction, as that would imply that >98% of the oxidized Hillsborough Soil (Table S1) corresponds to soluble species. Regardless, fractionating TOM samples in particulate and dissolved fractions would be of high importance in future oxidation experiments.
6. **Global carbon cycle amendments:** Following all experimental work and robust determination of how TOM degrades upon its exposure to degradation, it will be necessary to develop an amended global carbon multi-k cycling model. This can be done by incorporating the refined rate constant(s) for TOM degradation along the land-to-ocean continuum and using an optimized KIE value defining the extent of isotopic fractionation. In this way, global carbon cycle models will properly be corrected for the newly discovered alteration of TOM to appear marine-like through oxidative camouflaging. Additionally, recommendations for amended mixing models should be also developed so future studies can accurately quantify TOM and OOM fractions considering that TOM oxidation had occurred.

## Supporting Information Available

Experimental protocol and conditions; description of kinetic modeling; model results; raw elemental and isotopic data; description of NMR spectroscopic analysis; NMR spectra and integrals; estimates of biopolymeric classes; supplementary figures assessing the effect of iron on oxidation (PDF)
