# Supplementary material for: Oxidation Camouflages Terrestrial Organic Matter to Appear Marine-like
Source: Environ Sci Technol. 2025 Mar 14;59(11):5607–20. doi: 10.1021/acs.est.4c12913 (PMC11948483; doi:10.1021/acs.est.4c12913)
Supplement: Supplementary file 1 — es4c12913_si_001.pdf [file es4c12913_si_001.pdf]

Oxidation camouflages terrestrial organic matter to appear marine-like

Aleksandar I. Goranov[a], Susan J. Carter[b], Ann Pearson[b*], Patrick G. Hatcher[a,b*]

[a]Department of Chemistry and Biochemistry, Old Dominion University, Norfolk, VA 23529 United States

[b]Department of Earth and Planetary Sciences, Harvard University, Cambridge, MA 02138 United States

*Corresponding authors. Emails: phatcher@odu.edu and apearson@eps.harvard.edu

Supplemental Information

Summary: 22 pages, 13 figures, 15 tables.

Table of Contents

Section 1. Experimental protocol and conditions (Figures S1-S3) 2

Section 2. Kinetic modeling and results (Figures S4-S6; Tables S1-S9)....................7

Section 3. Assessment of total iron as a driver for oxidation (Figure S7)...............15

Section 4. NMR characterization (Figures S8-S13; Tables S10-S15) 16

References 22

Section 1. Experimental protocol and conditions

Experimental protocol. Prior to experimentation, 20-mL glass vials, glass stir rods, mortars, and pestles were acid-cleaned and combusted. Terrestrial organic matter (TOM) samples were dried at 105 ^{O}C for 12 hours to ensure complete dryness. After equilibration to room temperature, seven vials were accurately weighed, and then about 2.0 grams (accurately weighed) of soil sample were added to each vial. 10 mL of 1M H_2O_2 were added to six of the vials (labeled T1, T2, T3, T3, T5, and T6) and 10 mL of ultrapure H_2O are added to the last vial (labeled as C = control). The suspensions were agitated using glass rods to ensure homogeneous distribution of the liquid reagent. Vials were placed in a dark fume hood and were covered with a large KimWipe. After 48 hours, vials were transferred to an oven set at 105 ^{O}C. The dried seven samples were then accurately weighed (after equilibrating to room temperature) to determine mass losses. After this the control and T1 samples were capped and stored until further processing.

To the remaining samples (T2, T3, T4, T5, and T6) 10 mL of 1M H_2O_2 were added again for a second cycle of oxidation. After 48 hours, samples were dried in the oven, weighed, and T2 was capped and stored until further processing. To the remaining samples (T3, T4, T5, T6) 10 mL of 1 M H_2O_2 were added again for continuing the oxidation with another cycle. This process was repeated until sample T6 had experienced six oxidation cycles. Each cycle is comprised of 48-hour active oxidation and 12-hour drying (estimated total reaction time of 60 hours).

After the seven vials had undergone their experimental paths (Figure S1) they were homogenized using mortars and pestles and then analytically characterized via elemental, isotopic, and spectroscopic methods (see main text for details).

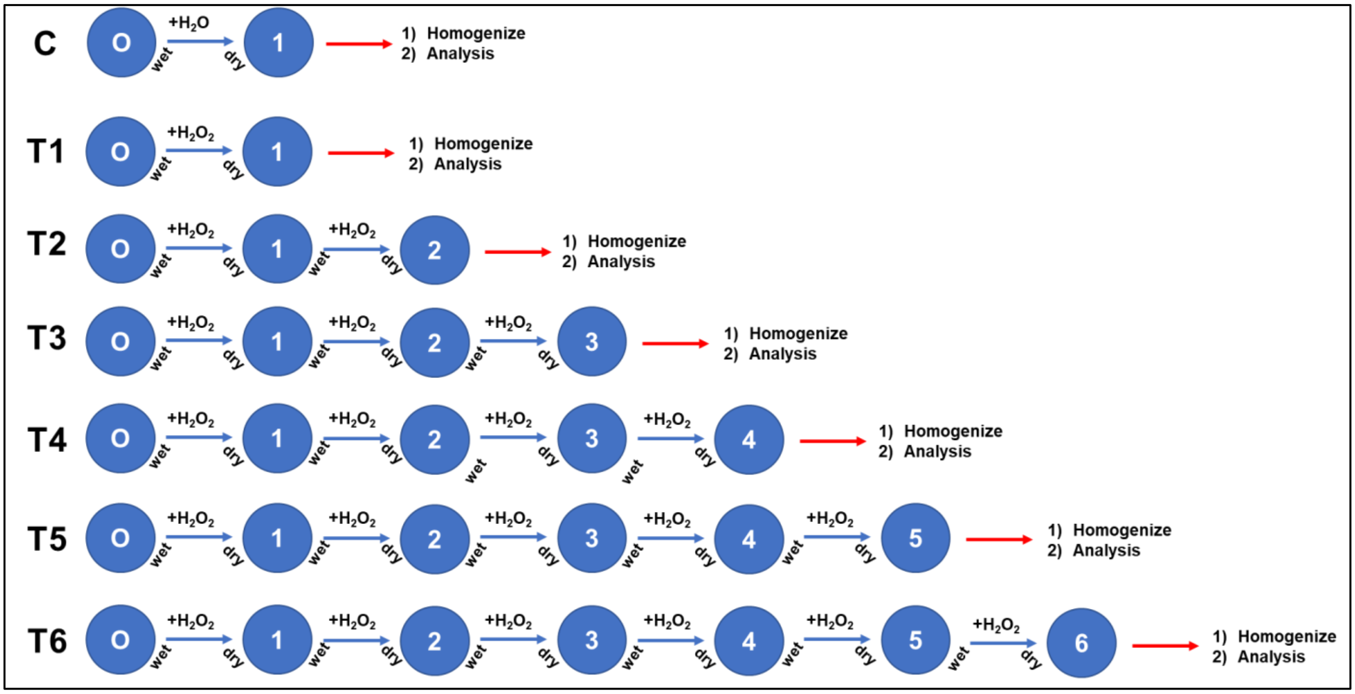

Figure S1. Experimental protocol scheme. Blue numbered circles in horizontal lines represent the oxidation path of each sample. Blue arrows indicate active oxidation time with 10 mL of 1 M reagent (H_2O_2 or H_2O) whereas red arrows indicate that a sample was capped and stored away prior to homogenization and analytical characterization.

Justification for temperature choice (105 ^{O}C). Heating samples at 105 ^{O}C was necessary to ensure complete dryness prior to elemental, isotopic, and spectroscopic analyses[1-3] though it must have led to losses of volatile carbon species. However, considering that particulate environmental matrices are commonly dried overnight at 105 ^{O}C prior to elemental (e.g., for total organic carbon, TOC), isotopic, and spectroscopic analyses, and that dissolved organic carbon measurements are commonly reported as non-purgeable organic carbon (NPOC), losses of volatile species is a common limitation in biogeochemical research and thus, our results are comparable to those in other publications and fit the analytical framework employed for developing global carbon cycle models. Heating at 105 ^{O}C also could lead to a minor heat-driven oxidation,[4] however, this is likely negligible relative to peroxide-driven oxidation. If had occurred, heat-driven oxidation contributed to the experimental design given that this study aimed test how TOM behaves under a strong oxidation gradient achieving > 90% of carbon losses.

Control experiments of four soils were performed to evaluate the effect of heating: 2 g of soil were suspended in 10 mL ultrapure water, and after 48 hours the water was evaporated at 105 ^{O}C for 12 hours. While sample weight was lost, all measurements were within the 2% uncertainty ranges (Figure S2A). Pahokee Peat lost the most weight agreeing with previous findings that peats lose more weight upon drying than mineral-rich soils.[4] Upon multiplication with TOC measurements the uncertainties increase, diminishing the weight loss trends (e.g., Elliott Soil's carbon weight increases; Figure S2B). C/N ratios uniformly increase by no more than 1.5 (Figure S2C). The δ^{13}C values change without a clear trend and values are within the propagated uncertainty values of 0.5 ‰ (Figure S2D). In summary, sample processing at 105 ^{O}C leads to negligible changes to the quantitative metrics employed in this study.

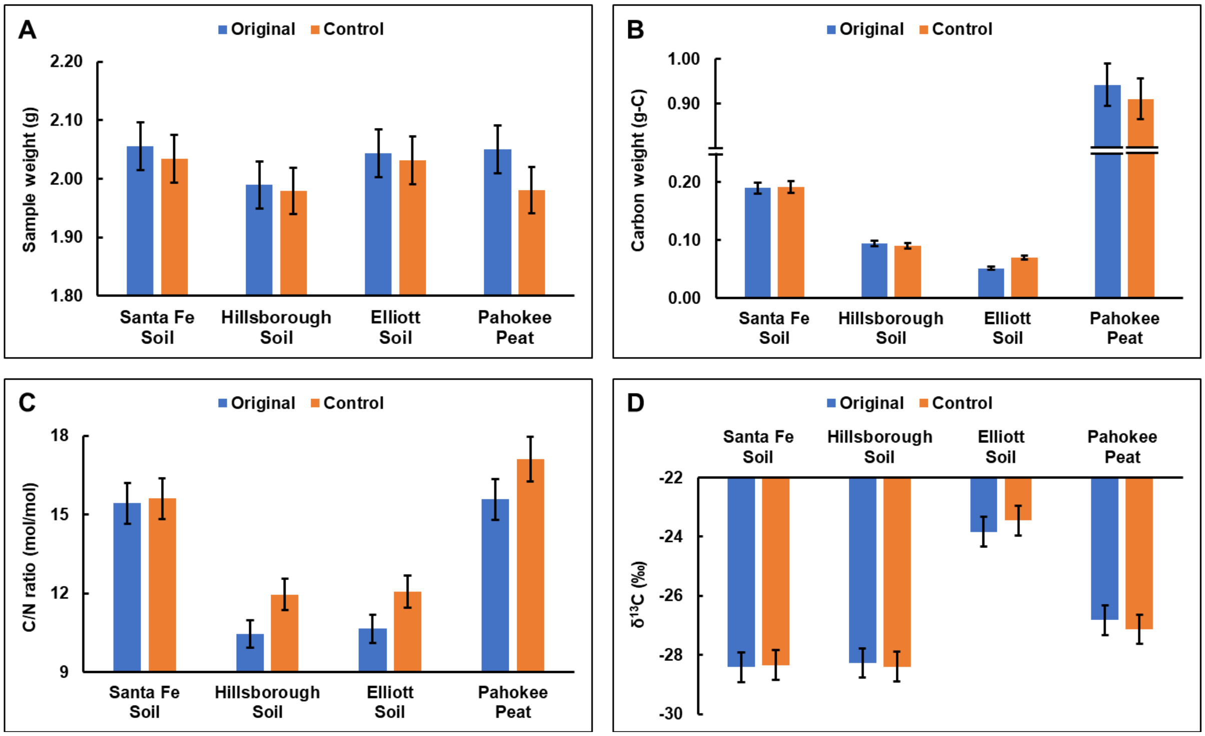

Figure S2. Elemental and isotopic results for ancillary control experiments showing differences among original TOM samples (blue bars) and control samples (orange bars). Error bars for sample weights (panel A) represent 2% propagated uncertainties, whereas error bars for sample carbon (panel B) and C/N ratios (panel C) represent 5% propagated uncertainties (as more two measurements were used in the calculation of the displayed metric). Error bars for δ^{13}C measurements represent propagated uncertainty values of 0.5 ‰.

Justification for reagent concentration choice (10 mL of 1 M H_2O_2). The volume of reagent was chosen to sufficiently suspend 2 grams of particulate soils into mixtures and ensure all organic matter is sufficiently and homogeneously wetted. The high concentration of 1 M was chosen for three reasons as explained below:

1. **Ensuring pseudo-first-order kinetics.** Environmental degradation generally follows pseudo-first order kinetics. This kind of kinetics are a special case of second-order kinetics (i.e., two substrates reacting together). For simplicity, the oxidation of carbon (C) by H_2O_2 is represented as:

$$\mathrm{C} + \mathrm{H_2O_2} \rightarrow \mathrm{Product} \qquad \text{Eq. S1}$$

The reaction rate for such two-component system can be described as shown below (t = time):

$$\mathrm{Rate} = -\frac{\mathrm{d[C]_t}}{\mathrm{dt}} = -\frac{\mathrm{d[H_2O_2]_t}}{\mathrm{dt}} = +\frac{\mathrm{d[Product]_t}}{\mathrm{dt}} \qquad \text{Eq. S2}$$

The rate of disappearance of C at a timepoint t would be described as shown below:

$$\mathrm{Rate} = -\frac{\mathrm{d[C]_t}}{\mathrm{dt}} = -\mathrm{k[C]_t[H_2O_2]_t} \qquad \text{Eq. S3}$$

Pseudo-first order kinetics would be observed when 1) one of the reactants is in too high concentration (i.e., $[C]_0 >> [H_2O_2]_0$ or $[C]_0 << [H_2O_2]_0$), 2) when both reactants are of equivalent concentrations (i.e., $[C]_0 = [H_2O_2]_0$), or 3) one of the reactants remains at constant concentration (i.e., either $[C]_0$ = constant or $[H_2O_2]_0$ = constant). As environmental oxidation occurs ubiquitously in soil, fluvial, and oxic sedimentary environments, the concentration of H_2O_2 (which in these equations represents oxidation) can be assumed to be constant (i.e., H_2O_2 is at a steady-state concentration, $[H_2O_2]_{ss}$). Experimentally this was mimicked by replenishing the H_2O_2 at each time point allowing C to be constantly exposed to H_2O_2. Therefore, it can be assumed that that $[H_2O_2]_t \approx [H_2O_2]_{ss}$, which can be then combined with the second-order rate constant k to yield a pseudo-first-order rate constant k' as shown below:

$$\mathrm{Rate} = \frac{\mathrm{d[C]_t}}{\mathrm{dt}} = [\mathrm{k} \times [\mathrm{H_2O_2}]_{ss}][\mathrm{C}]_t = \mathrm{k'[C]_t} \qquad \text{Eq. S4}$$

The integrated rate law for Eq. S4 is then equal to:

$$\mathrm{C_t}/\mathrm{C_0} = e^{-k't} \qquad \text{or} \qquad \ln\left(\mathrm{C_t}/\mathrm{C_0}\right) = -k't \qquad \text{Eq. S5}$$

Often these pseudo-first-order rate constants are labeled as “apparent”, because this is what is directly measured analytically. For simplicity, they are labeled as k and not k' (which labeling will be used hereafter). Considering that organic matter is comprised of multiple fractions of different lability, environmental kinetics are often modeled to describe the degradation of multiple components that degrade following pseudo-first-order kinetics. Eq.

S6 shows the degradation of carbon in a system where a labile fraction (f_{lb}) and a refractory fraction (f_r) degrade with two different rate constants (k_{lb} and k_r, respectively)

$$C_t/C_0 = \left[f_{lb} \times e^{-k_{lb}t}\right] + \left[f_r \times e^{-k_r t}\right] \quad \text{Eq. S6}$$

In summary, choosing 1M H_2O_2 was necessary to ensure that the oxidation reagent was not exhausted during the duration of the experiment and that pseudo-first-order kinetics were sustained. If lower concentrations were used, the experimental data would have started to follow true second-order-kinetics behavior. Characterizing such systems would have been challenging as it would have required the quantification of various carbon fractions (particulate carbon, dissolved carbon, etc.) as well as of various reactive species (hydroxyl radicals, peroxyl radicals, superoxide, etc.). Performing such measurements was out of the scope of this work as this study aimed to test how consistent exposure to oxidation altered TOM signatures.

2. **Ensuring no microbial contamination.** Given the evolution of gaseous species (CO_2, CO, etc.) during the oxidation it was necessary to keep the vials open. While precautions against microbial contamination were taken (drying at 105 ^{O}C, performing experiments in a fume hood with strong filtered-air flow), the possibility of microbial contamination remained. The use of biocide (e.g., $HgCl_2$, NaN_3) was considered, but avoided as the use of such compounds could further complicate the experiments, might affect kinetic trends, and could also affect following instrumental analyses (e.g., using NaN_3 would invalidate C/N ratio measurements). A strong oxidizing environment produced from the 1 M H_2O_2 would have eliminated any microbial contamination if such had occurred.

3. **Practicality.** Simulating environmental oxidation in laboratory conditions is incredibly challenging as environmental oxidation occurs in long geologic timescales, which are impractical to reproduce. Thus, accelerated laboratory experiments are commonly employed. For example, in photochemical oxidation studies employing solar simulation techniques it is common that experimentation length is significantly shortened (e.g., Bostick et al.[5] performed 32-day long laboratory irradiations equivalent to 76.8 environmental days). In our study, the employment of strong peroxide concentrations parallels this approach and in six oxidation cycles (equivalent of 15 laboratory days) it was possible to achieve oxidation that would likely occur on the timescale of environmental months to years. Unfortunately, at present, it is not possible to quantify the exact equivalency, and thus, the timescale of our study remains arbitrary. Therefore, the reported rate constants (Table S1) should be considered only on a relative basis (i.e., to compare the degradability of the labile and refractory components of different TOM samples). These rate constants should not be used in global carbon cycle multi-k models[6, 7] before their proper rescaling to environmental kinetic rates, which will be done in future studies.

To exemplify the necessity of using 1 M H_2O_2, three soils were oxidized using 0.1 M H_2O_2 (Figure S3). The results showed that the lower concentration of peroxide was insufficient to obtain significant carbon degradation (i.e., C_t/C_0 did not go below 50 %) and isotopic fractionation was only about 1 ‰. Larger carbon degradation and isotopic fractionation would have occurred if the 0.1 M H_2O_2 experiments were extended for longer periods of time, but this would have led to issues such as the kinetics deviating away from pseudo-first-order behavior, higher risk for microbial contamination, and poor practicality due to longer laboratory experimentation.

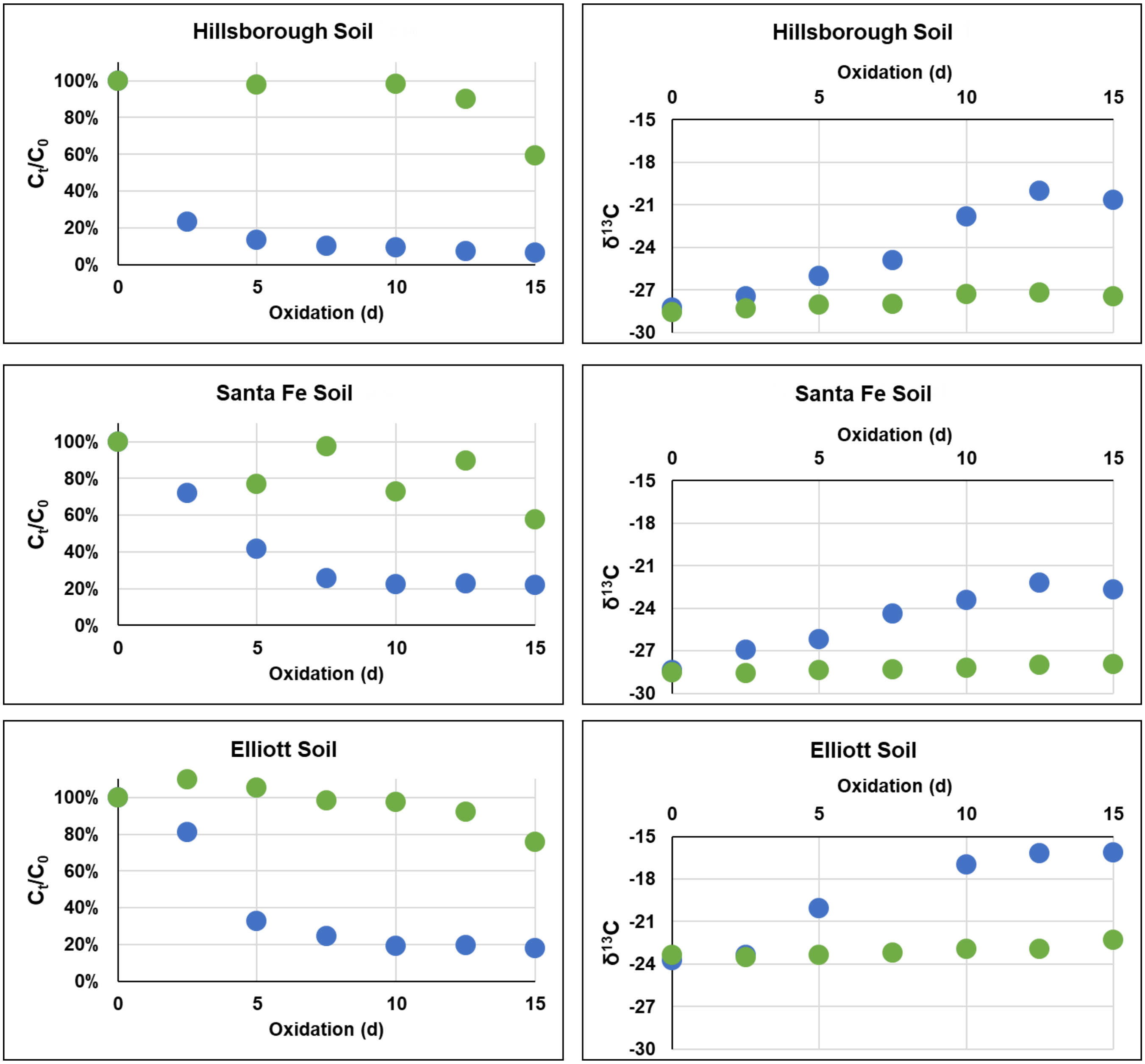

Figure S3. Remaining carbon after oxidation (C_t/C_0, left panels) and stable carbon isotopic composition ($\delta^{13}C$, right panels) of three select samples (Hillsborough Soil - top panels; Santa Fe Soil - middle panels; and Elliott Soil - bottom panels) oxidized with 10 mL 1 M H_2O_2 (blue circles) and 0.1 M H_2O_2 (green circles) following the protocol on Figure S1. Please note that some values exceed 100 % of C_t/C_0 due to associated uncertainties (error bars not shown for clarity).

Section 2. Kinetic modeling and results

Data from oxidation time series, both the fraction of remaining carbon (i.e., C_t/C_0) and its isotopic composition (i.e., $\delta^{13}C$) were used for kinetic modeling of organic matter degradation rates. Models using both first-order (Eq. S5) and time-dependent power law approaches (Eq. S7) were calculated. In the latter approach, k(t) is a rate constant whose value changes throughout the oxidation experiment as a power function of time t.[7] Except for the poorly degraded Pahokee, Okefenokee, and Dismal Swamp samples, fits to Eq. S7 show improvement relative to a constant value of k, indicating the reaction rate decreases as organic matter degradation progresses (Figure S4).

$$k(t) = k_{max} t^{b} \qquad \text{Eq. S7}$$

To model the evolution of carbon isotope ratios during the oxidation reaction, we define a kinetic isotope effect (KIE) for environmental oxidation. The KIE is a constant describing how a given process (here, oxidation) changes the ^{13}C content of organic matter as the reaction proceeds (Eq. 8). Note that the notation for $\delta^{13}C$ is simplified to δ.

$$\delta(t) = \delta_0 + \mathrm{KIE} * \ln\left({}^{C_t}/_{C_0}\right) \qquad \text{Eq. S8}$$

We assume that because the oxidation reaction conditions are applied in the same way to all samples, the KIE also is the same and constant throughout the experiments. Application of this KIE in a closed system containing a single endmember TOM source, in which the product is CO_2 or other volatile material with accumulation of leftover TOM, implies that $\delta^{13}C$ values of this residual TOM would evolve according to the contours in Figure S5.[8]

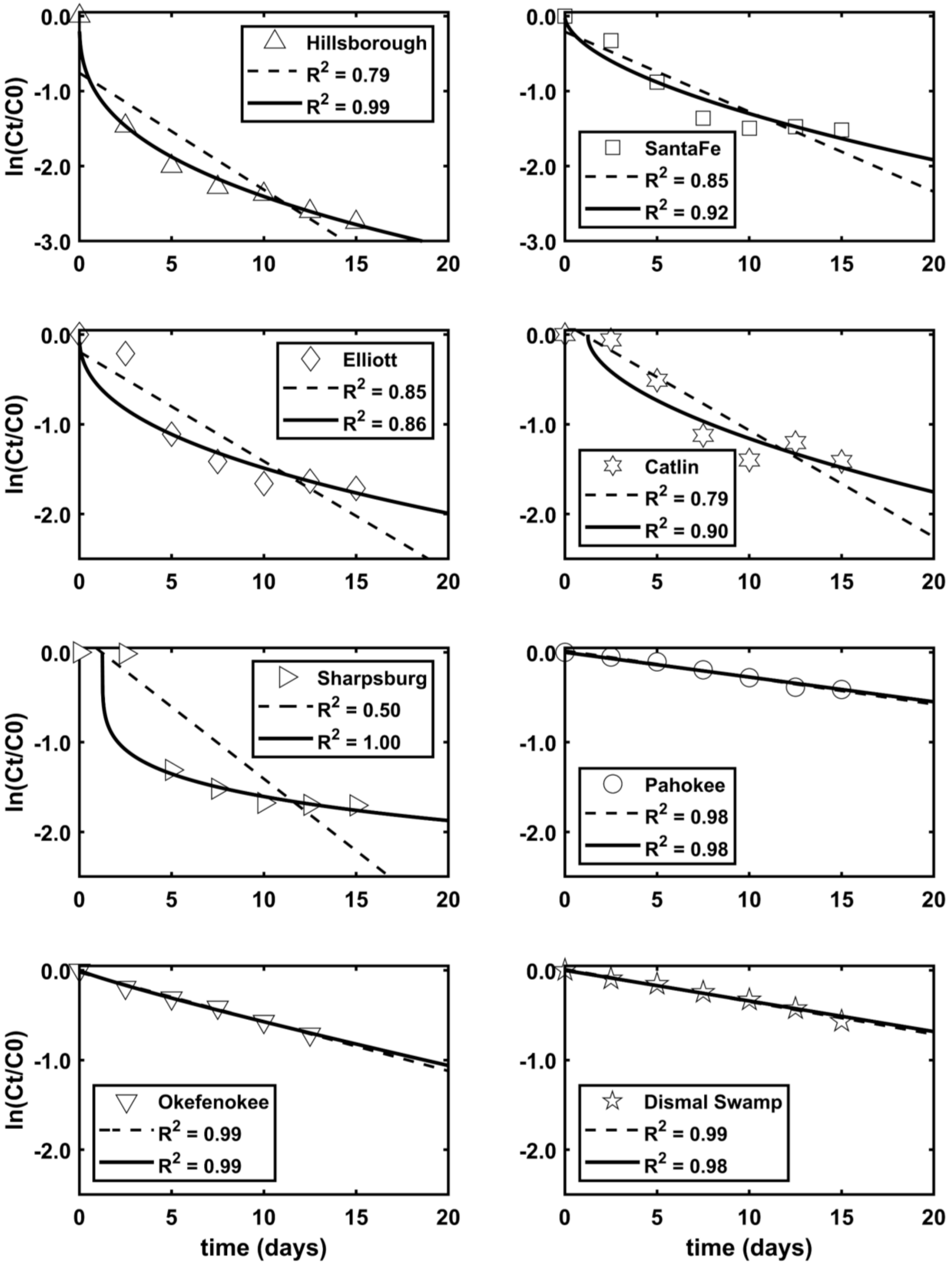

Figure S4. Linear (dashed lines, one-component degradation) vs. power-law (solid lines, multiple-component degradation) fits of oxidation-induced bulk organic carbon degradation in eight TOM samples; power-law results also are shown in the inserts of Figure 1 (main text). Error bars are not shown for clarity.

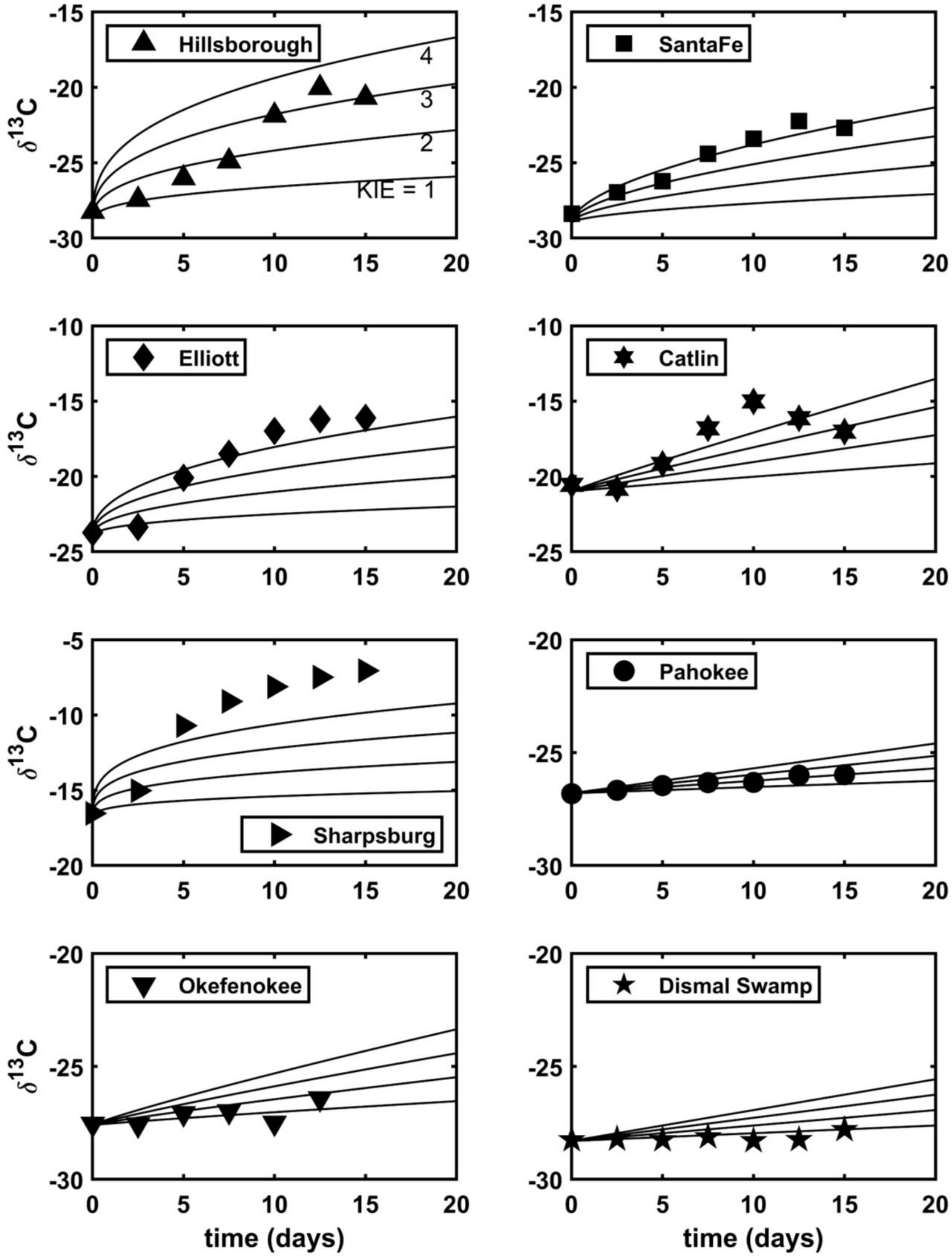

Figure S5. Disagreement between the hypothetical single-endmember degradation of TOM (shown as constant isolines representing kinetic isotope effect (KIE) values 1, 2, 3, and 4 ‰) vs. the observed data (solid symbols) which cross multiple KIE lines. Error bars are not shown for clarity.

The five samples that become significantly ^{13}C-enriched across the experiments (i.e., all except the three organic-rich peats) do not follow the KIE contours on Figure S5, implying that the oxidation is heterogeneous. As a possible explanation for this behavior, we explored the alternative of a mixed-endmember system having labile (f_{lb}) and refractory (f_r) fractions (as shown on Eq. S6). This mass balance approach was combined with the KIE framework (Eq. S8) yielding Eq. S9 (also shown in the main text as Eq. 4).

$$\delta(t) = \frac{\left[f_{lb}\left(\frac{C_t}{C_0}\right)_{lb}\left(\delta_{lb_0} + \mathrm{KIE}\cdot k_{lb}t\right) + f_r\left(\frac{C_t}{C_0}\right)_r\left(\delta_{r_0} + \mathrm{KIE}\cdot k_r t\right)\right]}{\left[f_{lb}\left(\frac{C_t}{C_0}\right)_{lb} + f_r\left(\frac{C_t}{C_0}\right)_r\right]}$$

Eq. S9
&
Main text Eq. 4

This equation was fitted to the data of the study with the following considerations:

- Mass balance approach:
  - Labile carbon fraction f_{lb} and refractory carbon fraction $f_r = 1 - f_{lb}$.
  - Assume f_r accumulates from f_{lb}.
  - Set $k_{lb} = k_{max}$ = early slope from $\ln\left({}^{C_t}/_{C_0}\right) = -kt$.
- Assumptions:
  - $k_r \leq$ late slope of $\ln\left({}^{C_t}/_{C_0}\right) = -kt$.
  - $\delta_{lb} \geq -33$ ‰.
  - $\delta_{t_r} \leq \delta_r \leq \left(\delta_{t_{max}} + 5\text{ ‰}\right)$.
- Unknowns: f_{lb}, δ_{lb_0}, δ_{r_0}, and KIE

Results of Monte Carlo resampling trials to obtain best-fit answers indicated that best model solutions were obtained for KIE values of 1 ‰ and 2 ‰. These two sets of solutions were statistically similar per cost function analysis implying that either result is equally likely to represent the KIE of oxidation. Thus, both solutions are provided in Table S1 to show the associated uncertainty with model results. The Pahokee, Okefenokee, and Dismal Swamp peats were not modeled with this two-endmember system, given their small fractional loss of carbon and corresponding minimal isotope shifts of 0.4 – 1.1 ‰.

A major limitation of this model is that it does not distinguish whether the small amount of f_r in the samples was present originally, or whether it was formed during the degradation of labile material. This affects the choice of appropriate control time-point, i.e., the timing of when the refractory organic carbon starts to break down. However, because the quantity of f_r is small and degrades at least an order of magnitude more slowly than the labile material (f_{lb}), any error in assigning its initiation (i.e., "t = 0 days" of breakdown) is also small and has likely led to minimal errors in model solutions.

Uncertainties associated with model constraints (e.g., the assumption of a two-fraction-only system, δ_0 initial ranges) and with estimated rate constants are challenging to quantify with the present data. A two-component model was selected based on the common division of organic matter pools into labile and refractory fractions, but this does not preclude other options. Differing reactivity according to chemical functional group (i.e., Figures S12 and S13), and/or a combination of both age-mixing and heterogeneous reactivity would increase the complexity of isotopic trajectories. This could lead to monotonic, plateau-like, or both positive and negative slope-changing behavior depending on sample types or depositional settings as modeled and shown in Figure S6. Further work with more constrained model systems, including specified

molecular classes of materials (e.g., lignin phenol compound mixtures), will be necessary to robustly test the assumptions and chosen constraints of the current model.

Table S1. Carbon isotopes and oxidation kinetic rates from optimization of a two-component model (Eq. S9). The upper value for k_r is the initial estimate (k_{r_est}), while the lower value is the optimized fit (k_{r_fit}). Answers for f and δ parameters are for fitting with KIE = 1 ‰ (upper value, **in blue**) or 2 ‰ (lower value, **in green**); both model solutions had statistically similar cost function values. A two-component system was not modeled for the Pahokee, Okefenokee, and Dismal Swamp peats due to the small observed change in $\delta^{13}C$.

Sample	Final TOC (wt.%)	Initial $\delta^{13}C$ (‰)	Final $\delta^{13}C$ (‰)	$k_{lb} = k_{max}$ (day^{-1})	k_{r_est} (day^{-1})<br>k_{r_fit} (day^{-1})	f_{lb}	f_r	δ_{lb} (‰)	δ_r (‰)
**Hillsborough Soil**	0.3	-28.3	-20.7	-0.400	-0.064<br>≤ -0.004	0.98<br>0.996	0.02<br>0.004	-28.8<br>-29.6	-19.8<br>-22.8
**Santa Fe Soil**	2.0	-28.4	-22.7	-0.177	-0.018<br>≤ -0.004	0.65<br>0.73	0.35<br>0.27	-32.3<br>-30.9	-21.1<br>-21.6
**Elliott Soil**	0.6	-23.7	-16.1	-0.223	-0.035<br>≤ -0.003	0.72<br>0.81	0.28<br>0.19	-27.2<br>-25.8	-15.0<br>-15.3
**Caitlin Soil**	0.72	-20.6	-17.0	-0.172	-0.027<br>≤ -0.001	0.73<br>0.72	0.27<br>0.28	-23.8<br>-23.7	-14.4<br>-15.2
**Sharpsburg Soil**	0.26	-16.5	-7.1	-0.250	-0.023<br>≤ -0.001	0.65<br>0.69	0.35<br>0.31	-19.1<br>-19.8	-6.7<br>-6.8
**Pahokee Peat**	41.7	-26.8	-26.0	-0.030	n/a<br>n/a	n/a	n/a	n/a	n/a
**Okefenokee Peat**	39.2	-27.6	-26.4	-0.055	n/a<br>n/a	n/a	n/a	n/a	n/a
**Dismal Swamp Peat**	56.7	-28.3	-27.8	-0.036	n/a<br>n/a	n/a	n/a	n/a	n/a

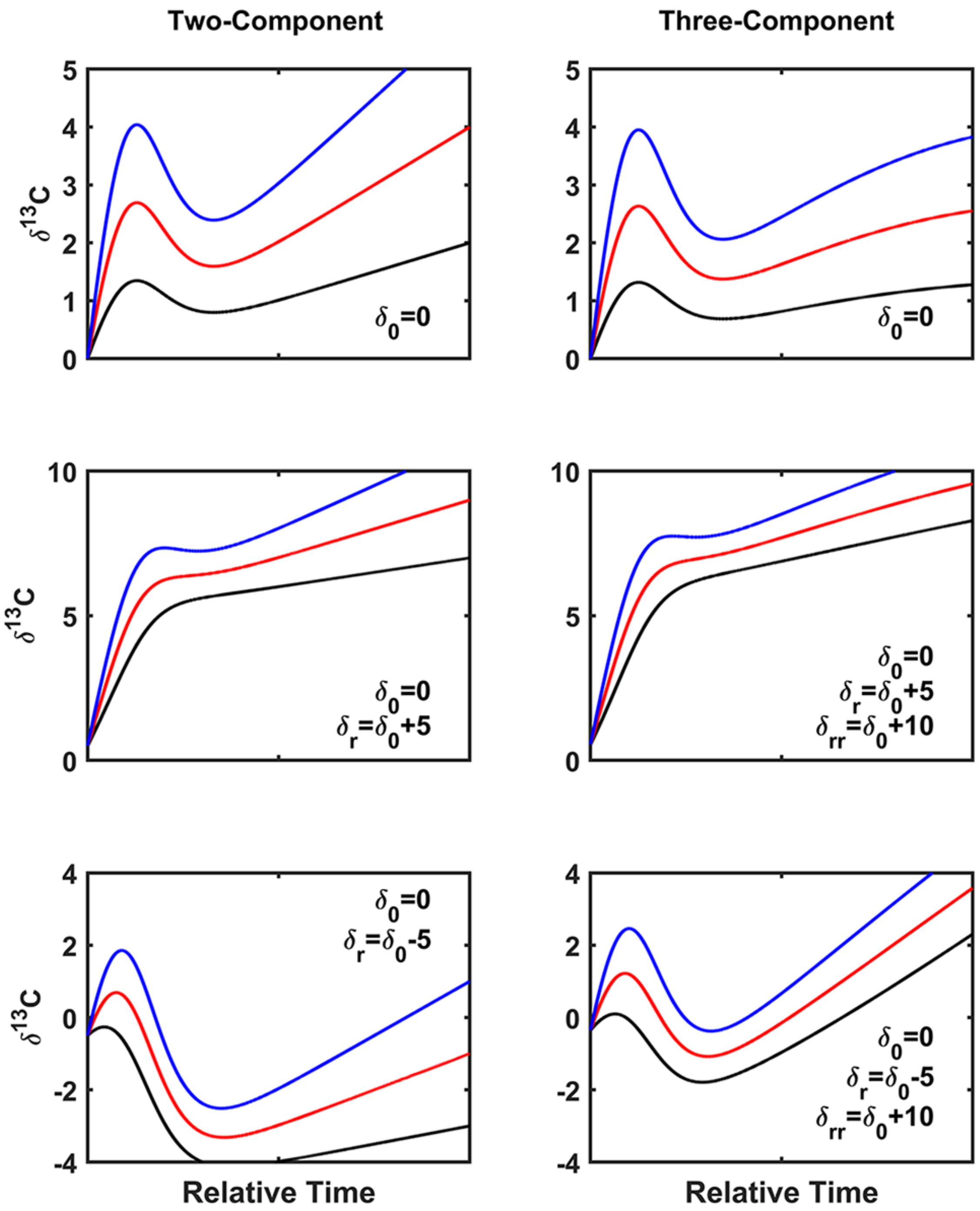

Figure S6. Hypothetical time-evolving isotope trajectories for two- (left panels) and three-component mixtures (right panels) with decreasing rate constants of degradation for the components. In the top panels, the value of δ_0 is equal for all components. In the middle panels, the value of δ_0 increases by 5 ‰ for each component. In the bottom panels, the value of δ_0 varies: labile fraction (f_{lb}) = 0 ‰, moderately refractory fraction (f_r) = δ_0 – 5 ‰, highly-refractory fraction (f_{rr}) = δ_0 +10 ‰. The rate constants for components f_{lb}, f_r, and f_{rr} are k, 0.1k, and 0.01k, respectively. The combination of heterogeneous endmember δ values with decreasing reaction rates can yield isotope trajectories that increase or decrease, and depending on initial composition may effectively terminate at final $\delta^{13}C$ values < $\delta(t_0)$ or > $\delta(t_0)$. Black, red, and blue lines are for KIE values of 1, 2, and 3 ‰, respectively.

Table S2. Elemental and isotopic data for the oxidation of Hillsborough Soil.

Time point	Oxidation (d)	TOC (%)	C_t/C_0 (%)	$\delta^{13}C$ (‰)
T0	0	4.49	100.0	-28.3
T1	2.5	1.08	23.1	-27.5
T2	5.0	0.64	13.5	-26.0
T3	7.5	0.48	10.2	-24.9
T4	10.0	0.44	9.3	-21.9
T5	12.5	0.35	7.4	-20.0
T6	15.0	0.31	6.4	-20.7

Table S3. Elemental and isotopic data for the oxidation of Santa Fe Soil.

Time point	Oxidation (d)	TOC (%)	C_t/C_0 (%)	$\delta^{13}C$ (‰)
T0	0	8.14	100.0	-28.4
T1	2.5	6.16	72.1	-27.0
T2	5.0	3.64	41.4	-26.2
T3	7.5	2.28	25.6	-24.4
T4	10.0	2.02	22.4	-23.4
T5	12.5	2.07	22.9	-22.2
T6	15.0	1.98	21.9	-22.7

Table S4. Elemental and isotopic data for the oxidation of Elliott Soil.

Time point	Oxidation (d)	TOC (%)	C_t/C_0 (%)	$\delta^{13}C$ (‰)
T0	0	3.04	100.0	-23.7
T1	2.5	2.47	80.9	-23.4
T2	5.0	1.03	32.9	-20.1
T3	7.5	0.77	24.3	Not Determined
T4	10.0	0.60	19.0	-17.0
T5	12.5	0.62	19.5	-16.2
T6	15.0	0.58	18.0	-16.1

Table S5. Elemental and isotopic data for the oxidation of Caitlin Soil.

Time point	Oxidation (d)	TOC (%)	C_t/C_0 (%)	$\delta^{13}C$ (‰)
T0	0	2.84	100.0	-20.6
T1	2.5	2.71	94.3	-20.8
T2	5.0	1.80	60.0	-19.2
T3	7.5	0.97	32.5	-16.8
T4	10.0	0.74	24.7	-15.0
T5	12.5	0.90	30.0	-16.1
T6	15.0	0.72	24.3	-17.0

Table S6. Elemental and isotopic data for the oxidation of Sharpsburg Soil.

Time point	Oxidation (d)	TOC (%)	C_t/C_0 (%)	$\delta^{13}C$ (‰)
T0	0	1.39	100.0	-16.5
T1	2.5	1.40	98.5	-15.0
T2	5.0	0.39	27.0	-10.7
T3	7.5	0.32	21.9	-9.1
T4	10.0	0.27	18.7	-8.1
T5	12.5	0.27	18.3	-7.5
T6	15.0	0.26	18.2	-7.1

Table S7. Elemental and isotopic data for the oxidation of Pahokee Peat.

Time point	Oxidation (d)	TOC (%)	C_t/C_0 (%)	$\delta^{13}C$ (‰)
T0	0	46.45	100.0	-26.8
T1	2.5	45.93	94.9	-26.7
T2	5.0	45.27	90.0	-26.5
T3	7.5	43.52	82.3	-26.3
T4	10.0	42.41	75.4	-26.3
T5	12.5	40.15	67.8	-26.0
T6	15.0	41.74	66.1	-26.0

Table S8. Elemental and isotopic data for the oxidation of Okefenokee Peat.

Time point	Oxidation (d)	TOC (%)	C_t/C_0 (%)	$\delta^{13}C$ (‰)
T0	0	50.9	100.0	-27.6
T1	2.5	45.6	82.6	-27.6
T2	5.0	43.9	73.4	-27.1
T3	7.5	43.1	66.2	-27.0
T4	10.0	40.0	56.5	-27.5
T5	12.5	39.2	49.0	-26.4
T6	15.0	Not Determined	Not Determined	Not Determined

Table S9. Elemental and isotopic data for the oxidation of Dismal Swamp Peat.

Time point	Oxidation (d)	TOC (%)	C_t/C_0 (%)	$\delta^{13}C$ (‰)
T0	0	59.45	100.0	-28.3
T1	2.5	58.29	91.0	-28.2
T2	5.0	59.49	85.7	-28.2
T3	7.5	59.08	78.3	-28.1
T4	10.0	59.41	71.9	-28.3
T5	12.5	59.58	65.2	-28.2
T6	15.0	55.84	56.7	-27.8

Section 3. Assessment of total iron as a driver for oxidation

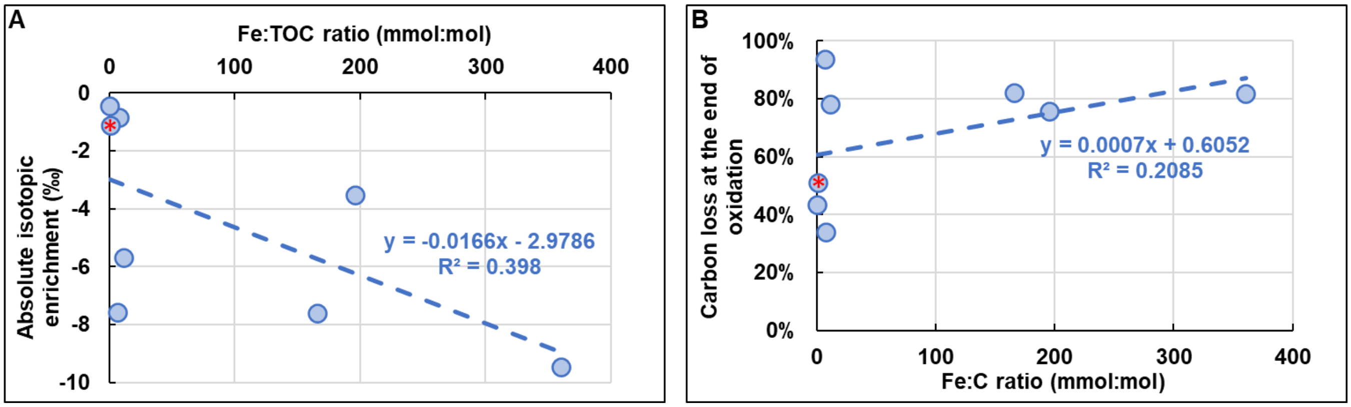

Figure S7. Correlational assessment of Fe:TOC ratios relative to the absolute isotopic enrichment (Final $\delta^{13}C$ - Initial $\delta^{13}C$; left panel) and carbon loss at the end of the oxidation (right panel). The data point labeled on the left panel with a red asterisk is of Okefenokee Peat, whose absolute isotopic enrichment is calculated as $\delta^{13}C$ at T5 - Initial $\delta^{13}C$ (sample T6 was not analyzed; see Table S8) whereas $\delta^{13}C$ at T6 was used for all other samples. Similarly, the data point labeled on the right panel with a red asterisk is calculated as 100 – C_5/C_0 whereas all other data points are calculated as 100 – C_6/C_0.

Section 4. NMR characterization

Solid-state ^{13}C NMR is the most powerful technique for characterizing organic matter in soils, sediments, and other environmental samples.[9] It is relatively insensitive and thus, a high TOC content is needed for obtaining spectra of acceptable signal-to-noise. The presence of minerals, and particularly iron-bearing ones, deteriorates the spectral quality. This can be circumvented in several ways such as treatment with hydrofluoric acid.[10] Such treatment was not done here to preserve the organic carbon in its native state given the pioneering nature of this study. Thus, spectra of acceptable quality were only acquired on the Santa Fe (main text Figure 2) and Pahokee (Figure S8) samples. The Hillsborough Soil has low TOC%, yielding spectra of poor quality (Figure S9). The Elliott Soil, in addition to its low TOC%, is also rich in Fe (2 wt. % at T0), which further deteriorated the signal (Figure S10). Santa Fe Peat and Pahokee Peat spectra were integrated (Tables S10 and S12) and deconvoluted using the Baldock model[11] estimating the contributions of carbohydrate, protein, lignin, lipid, and char (Tables S11 and S13).

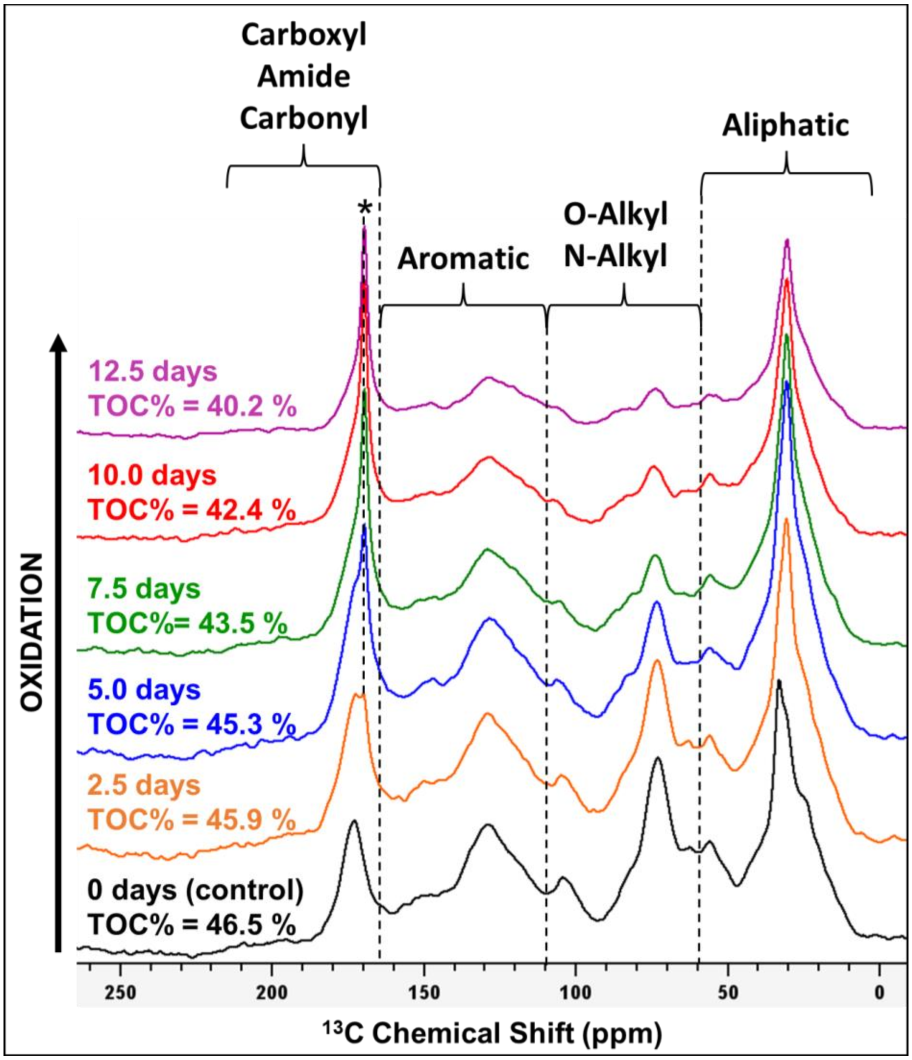

Figure S8. Solid-state ^{13}C NMR spectra of Pahokee Peat showing the loss of total organic carbon (TOC) with increasing oxidation. Aromatic (mainly from lignin), O-alkyl and N-alkyl (mainly from carbohydrates and proteins), and carboxyl signals diminish whereas the oxidized residue became gradually enriched in aliphatic remains as well as oxalate (labeled with an asterisk).

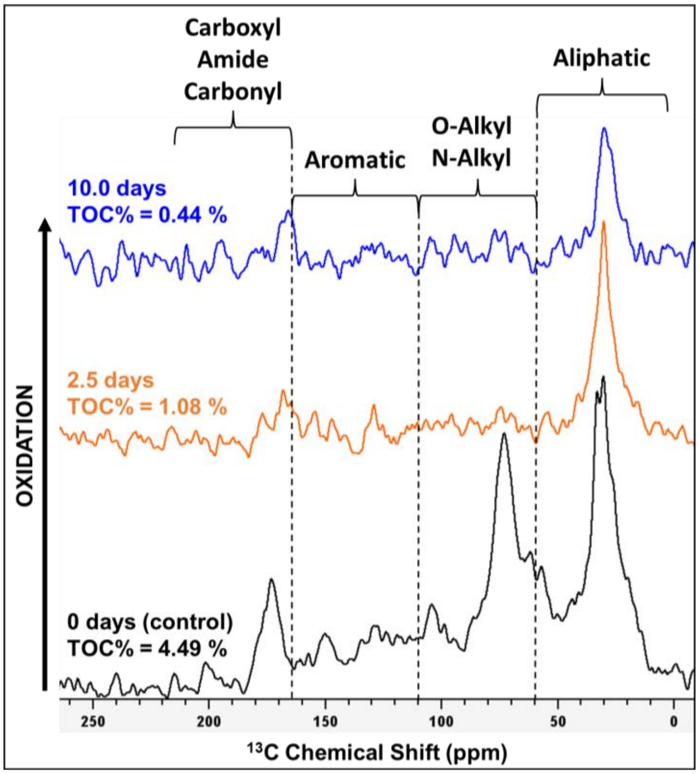

Figure S9. Solid-state ^{13}C NMR spectra of Hillsborough Soil showing the loss of total organic carbon (TOC) with increasing oxidation. Aromatic (mainly from lignin), O-alkyl and N-alkyl (mainly from carbohydrates and proteins), and carboxyl signals diminish whereas the oxidized residue became gradually enriched in aliphatic remains. The spectra become increasingly noisy due to the incredibly low TOC of the analyzed samples.

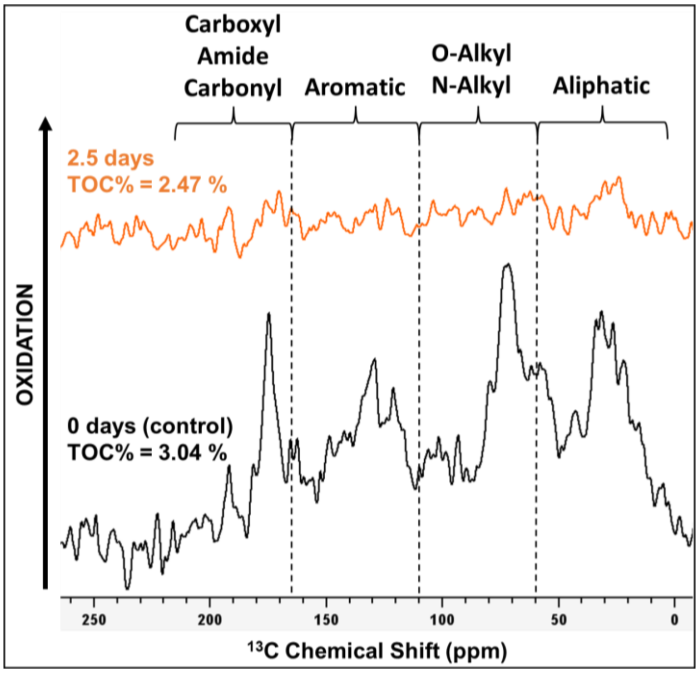

Figure S10. Solid-state ^{13}C NMR spectrum of Elliott Soil before and after oxidation. The spectra are incredibly noisy due to the low TOC and high concentration of iron limiting the ability to interpret the chemical changes occurring to this sample.

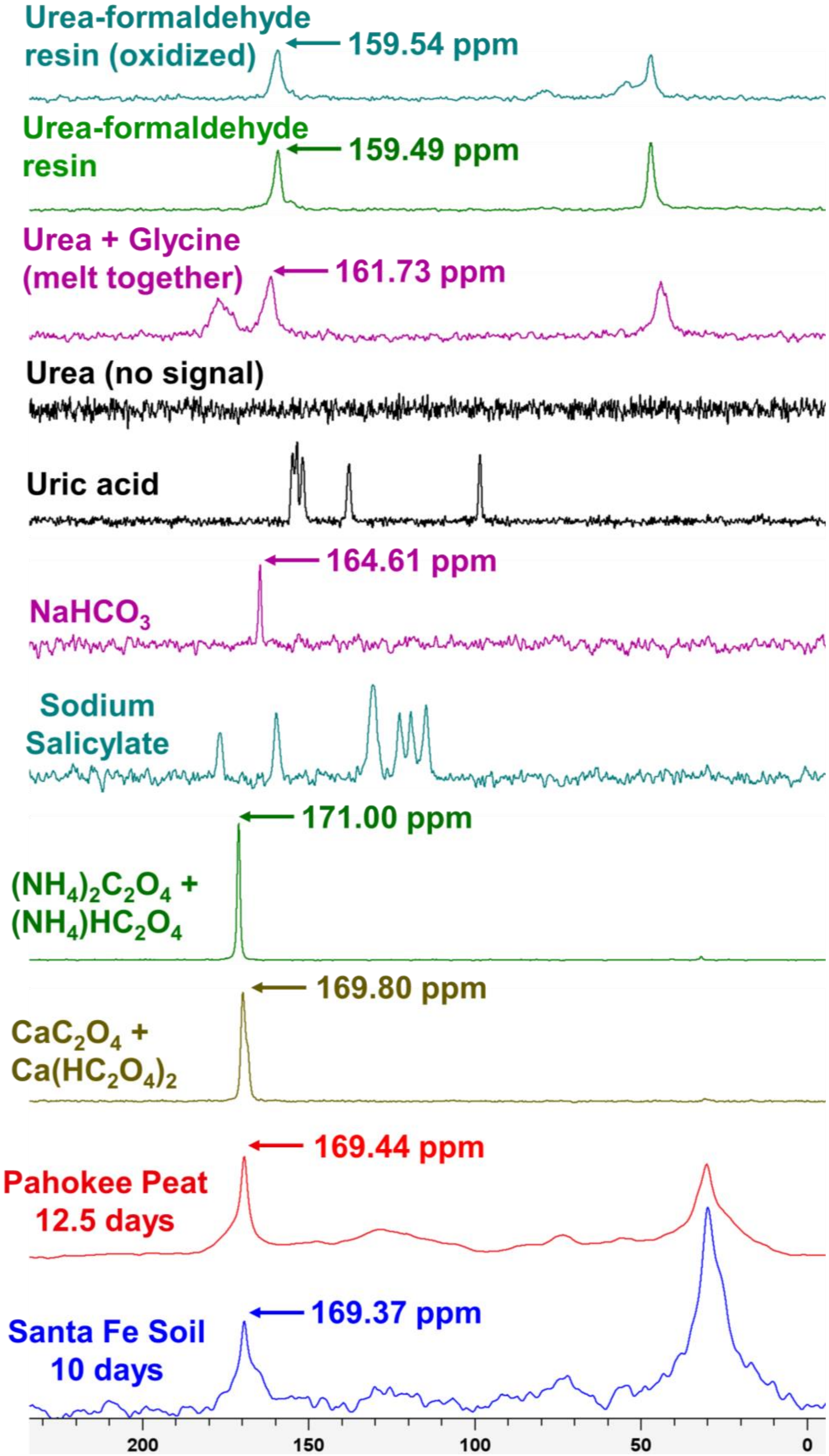

Figure S11. Comparison of solid-state ^{13}C NMR spectra of oxidized Pahokee Peat and Santa Fe Soil to spectra of standard compounds and materials for investigating the chemical nature of the sharp emergent peak at 169.4 ppm in oxidized TOM samples. All spectra are externally referenced to glycine to ensure chemical shift axis comparability.

Table S10. NMR integrals for the oxidized samples of Santa Fe Soil.

	0 – 45 ppm	45 – 60 ppm	60 – 95 ppm	95 – 110 ppm	110 – 145 ppm	145 – 165 ppm	165 – 215 ppm
T_0	31.38	9.24	27.13	6.69	12.27	4.58	8.72
T_1	40.08	8.71	18.12	4.35	11.54	5.14	12.05
T_2	43.85	6.99	16.10	3.09	11.47	5.28	13.21
T_3	50.01	6.41	11.96	2.39	8.19	5.73	15.30
T_4	56.64	6.08	11.97	1.30	7.73	5.64	10.64
T_5	N.D.	N.D.	N.D.	N.D.	N.D.	N.D.	N.D.
T_6	N.D.	N.D.	N.D.	N.D.	N.D.	N.D.	N.D.

Table S11. Estimated biopolymer contributions in the oxidized samples of Santa Fe Soil using Baldock modeling [11].

	Carbohydrate (%)	Protein (%)	Lignin (%)	Lipid (%)	Char (%)
T_0	25.62	20.85	19.76	28.89	4.87
T_1	13.32	17.12	21.19	42.02	3.13
T_2	10.25	8.46	19.47	51.24	4.43
T_3	4.35	2.07	21.64	62.25	0.00
T_4	3.45	0.00	19.31	72.26	0.00
T_5	N.D.	N.D.	N.D.	N.D.	N.D.
T_6	N.D.	N.D.	N.D.	N.D.	N.D.

Table S12. NMR integrals for the oxidized samples of Pahokee Peat.

	0 – 45 ppm	45 – 60 ppm	60 – 95 ppm	95 – 110 ppm	110 – 145 ppm	145 – 165 ppm	165 – 215 ppm
T_0	25.35	7.92	21.92	5.39	20.07	6.59	12.75
T_1	25.74	7.65	20.00	5.02	19.53	7.11	14.94
T_2	28.53	7.48	16.49	4.55	19.21	7.38	16.35
T_3	30.88	7.26	14.09	4.01	18.95	7.20	17.61
T_4	30.90	7.30	13.41	3.40	18.68	7.33	18.98
T_5	35.39	6.70	11.74	3.32	18.71	7.17	16.96
T_6	N.D.	N.D.	N.D.	N.D.	N.D.	N.D.	N.D.

Table S13. Estimated biopolymer contributions in the oxidized samples of Pahokee Peat using Baldock modeling [11].

	Carbohydrate (%)	Protein (%)	Lignin (%)	Lipid (%)	Char (%)
T_0	20.88	22.52	11.72	20.97	21.11
T_1	17.78	17.90	16.82	22.99	18.37
T_2	12.75	14.15	20.35	27.99	16.47
T_3	9.64	12.91	19.57	31.79	16.41
T_4	8.65	12.88	19.78	31.79	15.84
T_5	6.15	8.95	19.39	39.64	16.22
T_6	N.D.	N.D.	N.D.	N.D.	N.D.

N.D. indicates not determined.

Using the Biopolymer % estimates and the amount of remaining carbon at each oxidation point (C_t/C_0), remaining biopolymer-C at time t per original biopolymer-C (i.e., $Biopolymer_t/Biopolymer_0$) were determined for all classes (Tables S14, S15). Data were fit to first-order degradation fits (Eq. S5), i.e., $Biopolymer_t/Biopolymer_0 = e^{-kt}$ (Figures S12, S13). For the Santa Fe sample (Figure S12), the protein and carbohydrate fractions appear to degrade the fastest, followed by the char and lignin fractions. The lipid fraction degrades the slowest likely because lipids are simultaneously degraded (remineralized by oxidation) and produced (by loss of aromatic groups). For the Pahokee Peat sample (Figure S13), carbohydrates, proteins, and char moieties degraded, but lignin and lipids appear to be produced. This is possible because this soil is rich in char (21%, Table S13), and lipids are known to be produced during the oxidative degradation of char.[12] If this soil had been oxidized further, e.g., to bulk $C_t/C_0 << 50$ %, it is likely that pseudo-first-order degradation would have become apparent for all biopolymeric components.

Table S14. Loss of functional groups for Santa Fe Soil measured by solid-state ^{13}C NMR; % relative to T_0.

Santa Fe Soil	$Carb_t/Carb_0$ (%)	$Protein_t/Protein_0$ (%)	$Lignin_t/Lignin_0$ (%)	$Lipid_t/Lipid_0$ (%)	$Char_t/Char_0$ (%)
T_0, 0 days	100%	100%	100%	100%	100%
T_1, 2.5 days	37%	59%	77%	105%	46%
T_2, 5.0 days	17%	17%	41%	73%	38%
T_3, 7.5 days	4%	3%	28%	55%	0%
T_4, 10.0 days	3%	0%	22%	56%	0%

Table S15. Loss of functional groups for Pahokee Peat measured by solid-state ^{13}C NMR; % relative to T_0.

Pahokee Peat	$Carb_t/Carb_0$ (%)	$Protein_t/Protein_0$ (%)	$Lignin_t/Lignin_0$ (%)	$Lipid_t/Lipid_0$ (%)	$Char_t/Char_0$ (%)
T_0, 0 days	100%	100%	100%	100%	100%
T_1, 2.5 days	81%	75%	136%	104%	83%
T_2, 5.0 days	55%	57%	156%	120%	70%
T_3, 7.5 days	38%	47%	137%	125%	64%
T_4, 10.0 days	31%	43%	128%	115%	57%
T_5, 12.5 days	20%	27%	112%	128%	52%

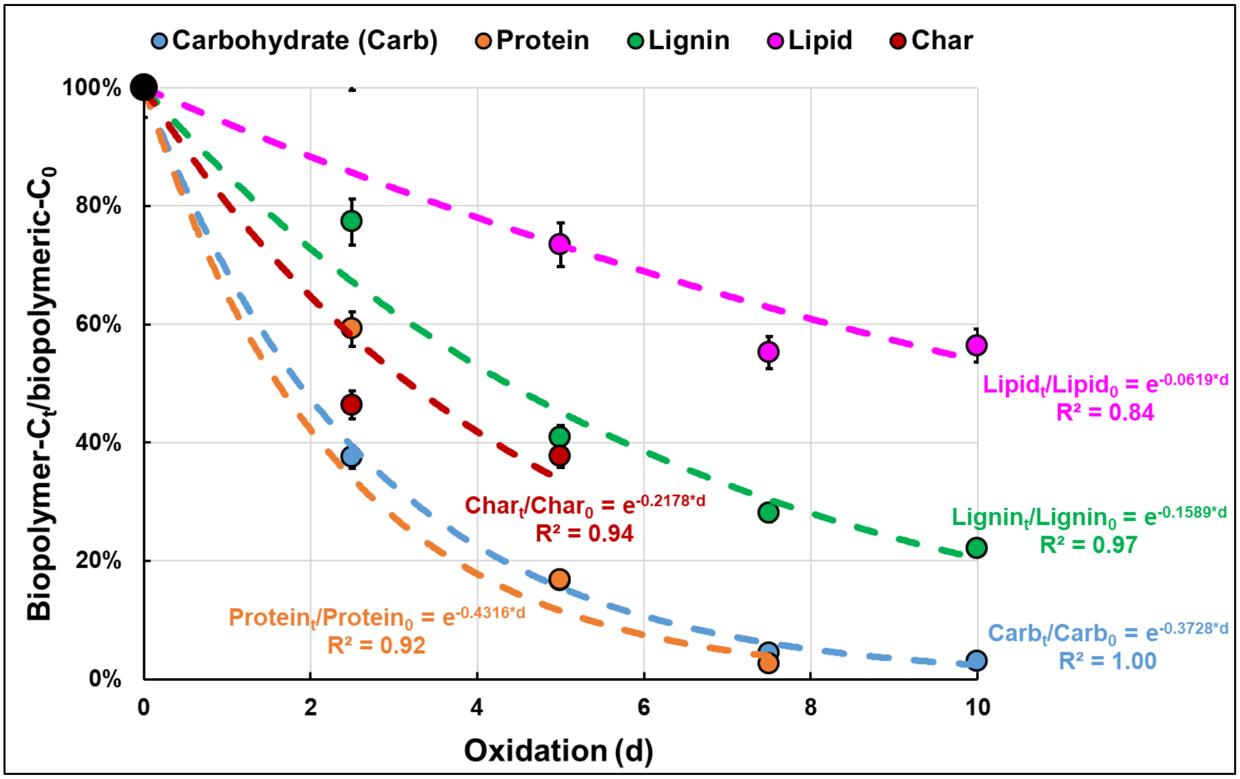

Figure S12. Oxidation curves of biopolymeric compounds found in Santa Fe Soil after oxidation with up to 10 days. Error bars correspond to a conservative propagated uncertainty of 10 %.

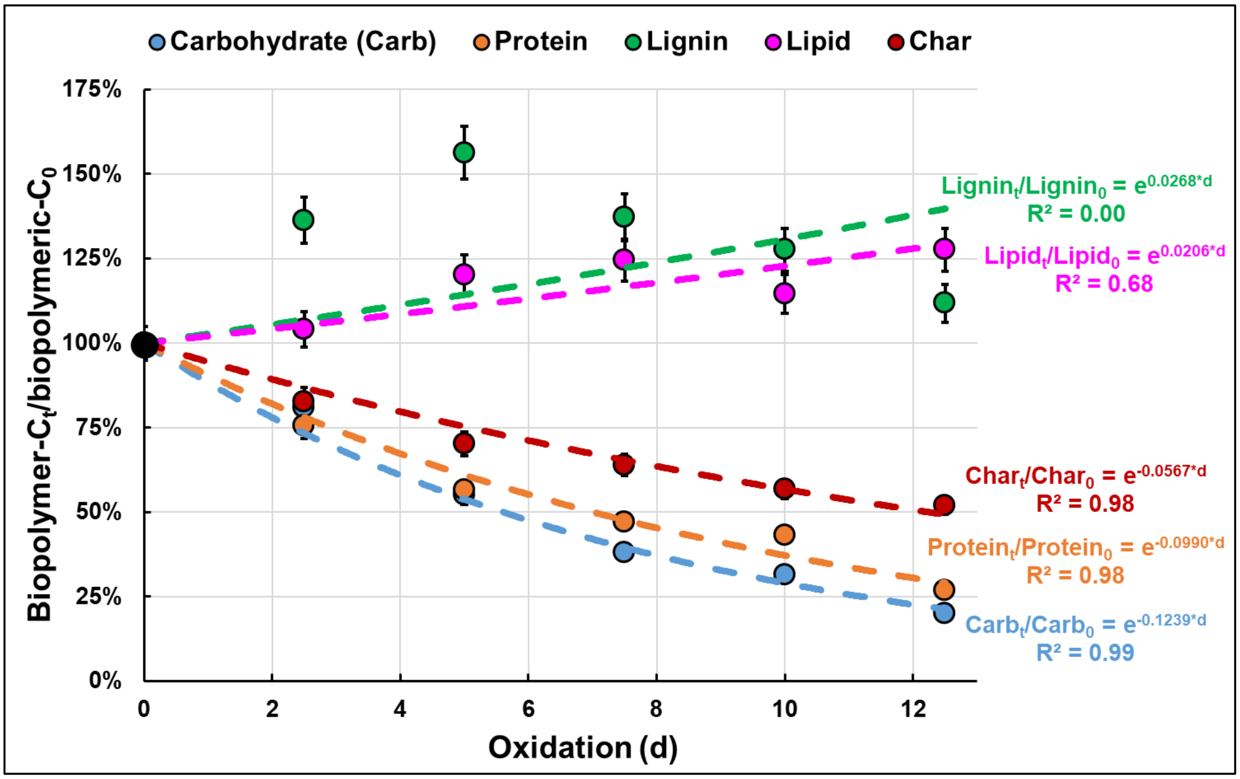

Figure S13. Oxidation curves of biopolymeric compounds found in Pahokee Peat after oxidation with up to 12.5 days. Error bars correspond to a conservative propagated uncertainty of 10 %.

References

(1) Hatcher, P. G.; Wilson, M. A. The effect of sample hydration on 13C CPMAS NMR spectra of fulvic acids. *Organic Geochemistry* **1991**, *17* (3), 293-299. DOI: 10.1016/0146-6380(91)90092-X.

(2) Leckrone, K. J.; Hayes, J. M. Water-Induced Errors in Continuous-Flow Carbon Isotope Ratio Mass Spectrometry. *Analytical chemistry* **1998**, *70* (13), 2737-2744. DOI: 10.1021/ac9803434.

(3) Laub, M.; Blagodatsky, S.; Nkwain, Y. F.; Cadisch, G. Soil sample drying temperature affects specific organic mid-DRIFTS peaks and quality indices. *Geoderma* **2019**, *355*, 113897. DOI: 10.1016/j.geoderma.2019.113897.

(4) O'Kelly, B. C. Oven-Drying Characteristics of Soils of Different Origins. *Drying Technology* **2005**, *23* (5), 1141-1149. DOI: 10.1081/DRT-200059149.

(5) Bostick, K. W.; Zimmerman, A. R.; Goranov, A. I.; Mitra, S.; Hatcher, P. G.; Wozniak, A. S. Photolability of pyrogenic dissolved organic matter from a thermal series of laboratory-prepared chars. *Science of the Total Environment* **2020**, *724*, 1-9. DOI: 10.1016/j.scitotenv.2020.138198.

(6) Berner, R. A. *Early Diagenesis: A Theoretical Approach*; Princeton University Press, 1980. DOI: 10.2307/j.ctvx8b6p2.

(7) Middelburg, J. J. A simple rate model for organic matter decomposition in marine sediments. *Geochimica et Cosmochimica Acta* **1989**, *53* (7), 1577-1581. DOI: 10.1016/0016-7037(89)90239-1.

(8) Mariotti, A.; Germon, J. C.; Hubert, P.; Kaiser, P.; Letolle, R.; Tardieux, A.; Tardieux, P. Experimental determination of nitrogen kinetic isotope fractionation: Some principles; illustration for the denitrification and nitrification processes. *Plant and Soil* **1981**, *62* (3), 413-430. DOI: 10.1007/BF02374138.

(9) Simpson, A. J.; Simpson, M. J. Nuclear magnetic resonance analysis of natural organic matter. In *Biophysico-chemical processes involving natural nonliving organic matter in environmental systems*, Senesi, N., Xing, B., Huang, P. M. Eds.; 2009; pp 589-650.

(10) Schmidt, M. W. I.; Knicker, H.; Hatcher, P. G.; Kögel-Knabner, I. Improvement of ^{13}C and ^{15}N CPMAS NMR spectra of bulk soils, particle size fractions and organic material by treatment with 10% hydrofluoric acid. *European Journal of Soil Science* **1997**, *48* (2), 319-328. DOI: 10.1111/j.1365-2389.1997.tb00552.x.

(11) Nelson, P. N.; Baldock, J. A. Estimating the molecular composition of a diverse range of natural organic materials from solid-state 13C NMR and elemental analyses. *Biogeochemistry* **2005**, *72* (1), 1-34. DOI: 10.1007/s10533-004-0076-3.

(12) Goranov, A. I.; Wozniak, A. S.; Bostick, K. W.; Zimmerman, A. R.; Mitra, S.; Hatcher, P. G. Photochemistry after fire: Structural transformations of pyrogenic dissolved organic matter elucidated by advanced analytical techniques. *Geochimica et Cosmochimica Acta* **2020**, *290*, 271-292. DOI: 10.1016/j.gca.2020.08.030.
